# The Influence of Each Facial Feature on How We Perceive and Interpret Human Faces

**DOI:** 10.1177/2041669520961123

**Published:** 2020-09-30

**Authors:** Jose A. Diego-Mas, Felix Fuentes-Hurtado, Valery Naranjo, Mariano Alcañiz

**Affiliations:** i3B—Institute for Research and Innovation in Bioengineering, Universitat Politecnica de Valencia, Valencia, Spain

**Keywords:** face perception, facial features appearance, facial traits, face judgments

## Abstract

Facial information is processed by our brain in such a way that we immediately make judgments about, for example, attractiveness or masculinity or interpret personality traits or moods of other people. The appearance of each facial feature has an effect on our perception of facial traits. This research addresses the problem of measuring the size of these effects for five facial features (eyes, eyebrows, nose, mouth, and jaw). Our proposal is a mixed feature-based and image-based approach that allows judgments to be made on complete real faces in the categorization tasks, more than on synthetic, noisy, or partial faces that can influence the assessment. Each facial feature of the faces is automatically classified considering their global appearance using principal component analysis. Using this procedure, we establish a reduced set of relevant specific attributes (each one describing a complete facial feature) to characterize faces. In this way, a more direct link can be established between perceived facial traits and what people intuitively consider an eye, an eyebrow, a nose, a mouth, or a jaw. A set of 92 male faces were classified using this procedure, and the results were related to their scores in 15 perceived facial traits. We show that the relevant features greatly depend on what we are trying to judge. Globally, the eyes have the greatest effect. However, other facial features are more relevant for some judgments like the mouth for happiness and femininity or the nose for dominance.

Humans have highly developed their ability to perceive faces and to process the information extracted from them (Bruce & Young, 1986; Damasio, 1985). The fusiform face area (Kanwisher et al., 1997) and the posterior superior temporal sulcus (Schobert et al., 2018) are a specialized neural network of our brains able to identify people; guess their gender, age, or race; or even, judge the emotions and intentions of the owners of the faces. Through this behavioral capacity to perceive faces, we use the facial appearance to make attributions such as personality, intelligence, or trustworthiness (Bruce & Young, 2012). Therefore, faces affect our everyday decisions (Little et al., 2007; Todorov, 2011; Todorov et al., 2008; Zebrowitz & Montepare, 2008) such as mate selection (Bovet et al., 2012; Dixson et al., 2016; Keating & Doyle, 2002), voting decisions (Little et al., 2007; Todorov et al., 2005), criminal justice decisions (Eberhardt et al., 2006; Wilson & Rule, 2015), or how social partners are chosen (Langlois et al., 2000). Due to the importance of appearance-driven judgments of faces, face perception has become a major focus not only for psychological research but also for neuroscientists, engineers, and software developers (Jack & Schyns, 2015).

Visual perception research has shown that the human brain processes faces in a different way to other kind of objects (Piepers & Robbins, 2012). *Part-based* perceptual models suppose that objects are processed on the basis of their components or parts (Biederman, 1987); although it is commonly agreed that this is the way in which we process most objects, faces are thought to be processed in a different way. In *relational* (Diamond & Carey, 1986) or *configural* (Bartlett et al., 2003) models of perception, basic face features are processed in a part-based way, however, the perception relies heavily on the variations in the positioning of and the spacing between these basic features. *Holistic* perceptual models integrate facial features into a gestalt whole when the human brain processes facial information (holistic face processing; Tanaka & Farah, 1993; Young et al., 1987). The holistic models do not exclude part-based processing from the global holistic face perception process (Mckone & Yovel, 2009; Rossion, 2008), and some part of the perception relies on part-based processing of faces.

The main objective of this work was to measure the effect size of each basic facial feature (e.g., two specifics eyes, a particular nose, a mouth, etc.) on the opinion of the observers about some of the whole face traits. Hereinafter, we will consider a face trait as any judgment that an observer can make about the physical characteristics of the face (e.g., attractiveness, masculinity/femininity, etc.) or about the emotional state of the face owner (e.g., sadness, happiness, fear, etc.). It is important to remark that we are considering each facial feature as a whole. For example, in this study, we consider the global appearance of the noses more than specific characteristics like dimensions or shapes.

Our secondary objective was to obtain models that predict the facial traits of faces from the combination of facial features that conform them. There are interactions between the facial features during face recognition tasks. However, regarding the facial traits assessment, although interactions between the features also exist, a more direct relationship with specific facial features can be established. For example, larger eyes, higher eyebrows, and smaller noses are perceived as baby-faced, and faces with some of these features are also perceived as baby-faced (Keating & Doyle, 2002; Zebrowitz & Montepare, 2008). A comprehensive discussion on this approach can be found in Zebrowitz-McArthur and Baron (1983). Although how the traits of a face are perceived depends on the whole face, the individual effect of each feature can explain part of the variation within the appraisals of the faces (Cabeza & Kato, 2000; Rakover, 2002). Accordingly, some studies have used additive models of the facial attributes appraisals which explains most of the feasible explained variance (Gill, 2017; Maloney & Dal Martello, 2006). Other studies have related individual facial features to perceptions of the targets’ personalities (Paunonen et al., 1999) or have predicted facial trait evaluations from facial features accurately (Rojas et al., 2011). Obviously, unexplained variation must remain due to the interaction between the features under consideration and because the facial features included in the models do not cover the whole face.

There are some previous works in this field (Brahnam & Nanni, 2010; Paunonen et al., 1999). In these studies, some specific characteristics of facial features are used as independent variables in the models (e.g., eye size, mouth fullness, nose width . . . ) or local techniques for face recognition are used. However, these approaches do not consider the global appearance of each feature or they consider characteristics that do not belong to the features themselves. Therefore, we propose a different approach to measure the effect size of each facial feature on observed traits and to build predicting models.

The different points of view in the face perception literature are reflected in the computational methods for analysis of facial information. A comparison of techniques shows different approaches to deal with faces (Rojas et al., 2011). Feature-based approaches automatically encode the geometry of faces using several significant points or areas and relationships between them, doing a metric or morphological assessment of the facial features. Examples of these kinds of techniques are those based on SIFT feature descriptors (Meyers & Wolf, 2008), point distribution models (Cootes et al., 2001), or local binary patterns (Ahonen et al., 2006). Image-based approaches rely on entire image of faces, considering all the information available, and encompassing the global nature of the faces. Image-based techniques include, for example, Fisherfaces (Belhumeur et al., 1997) or Eigenfaces (Turk & Pentland, 1991). Some work on facial features characterization has been done mixing feature-based and image-based techniques (Klare & Jain, 2010). Finally, artificial neural networks, support vector machines, and deep learning methods (Dizaji et al., 2017; Huang et al., 2014) are currently used for facial feature extraction, yielding good results (Xie et al., 2016).

The problem of relating facial information and social judgments is compound by the fact that the space of possible hypotheses (what features drive specific social perceptions) is infinitely large (Todorov et al., 2011). Conventional (or direct) approaches to obtaining perception models systematically manipulate the stimulus in order to achieve different responses. The stimulus–response relationships are obtained by correlating the attributes of those manipulated stimuli together with their corresponding responses. However, using these direct approaches is difficult when the number of independent variables (attributes) and the number of possible values of such variables are great. In the case of faces, the number of variables that must be used to describe the facial features that can drive social perceptions is huge. There are no clear definitions of the basic facial features such as eyes, noses, or mouths. For example, an eye could be a collection of smaller features (e.g., upper eyelid, lower eyelid, pupil, eyelash . . . ), and each of them can be described as a collection of lines, shadows, or surfaces. Even considering only a small number of features, the combination of all the possible feature values rapidly proliferate.

To face these problems, feature-based approaches use only specific attributes of the face such as the relationships among a small number of key points or regions of interest. This approach reduces the amount of information used to describe the face using only some attributes perceived to be relevant or descriptive of the face. These approaches establish relationships between face descriptors and the evaluated social trait. Whole image-based approaches, such as Fisherfaces or Eigenfaces, use appearance-based representations of faces, which encode all available information about a face in a few meaningful variables.

Our proposal is a mixed feature-based and image-based approach. We are interested in the size of the effect of the basic facial features on the perception of some social traits. In a first step, we establish a reduced set of relevant specific attributes to describe a face: the basic facial features. Then, we use a principal component analysis (PCA) on the facial features to encode all the available information about each feature in a few meaningful variables. Then, we use these variables to categorize the facial features by their global appearance. In this way, we are avoiding the problem of the standard definition of face parts. We consider that a basic facial feature, such as a mouth, is defined by all the attributes in the image of the mouth, and we are considering all of them simultaneously. Finally, we use these few meaningful variables obtained from the PCA to cluster the facial features by appearance. Features belonging to the same category or cluster have similar appearances and are supposed to have the same effect size on the face traits. In this way, it is possible to describe specific faces by the clusters to which their features belong. Using this procedure on a large set of faces, relationships between the clusters of the features and the traits perceived in the faces can be established.

## Method

### Selection of Faces, Facial Traits and Facial Features

To examine the influence of the appearance of each facial feature on the facial traits, we need a set of faces assessed by several observers with respect to the facial traits to be analyzed. On the other hand, the features of the faces must be classified by the similitude of their appearances.

Our faces express emotions by changing the shape of their features. Observers can judge if the observed person’s current emotional state is happy, angry, or sad based on these changes. For example, the owner of a smiling face looks happy but bored or tired if the face is yawning. Regardless of the expression, people make social trait inferences based on the facial appearance of faces in a neutral state. These inferences are not related to an instantaneous emotional state, although they are driven in part by their structural resemblance to emotional expressions (Petrican et al., 2014; Said et al., 2009). In this way, a neutral face can elicit in the observer sensations such as happiness, sadness, or dominance. The face’s owners can seem to be happy people although they are not smiling or laughing. In this work, we are interested in these facial traits that are not related to the instantaneous emotional state of the faces. For this reason, we used only neutral faces in our study, without expressions or deformations of the facial features.

Therefore, our first step was to obtain a set of photographs of faces with neutral expressions. After reviewing several well-known databases (Chihaoui et al., 2016), we selected the Chicago Face Database (Ma et al., 2015). This database contains high-resolution standardized images of real faces. The faces of the database belong to people between the ages of 18 and 40 living in the Chicago (U.S.A) area. All the images in the database have the same size and resolution; faces have the same position, pose, and orientation, and the background and illumination are uniform. The homogeneity of the conditions in which the images were obtained was an important factor to select this face database because, for example, differences in the contrast of the image (Russell, 2003) or pose (Åsli et al., 2017; Favelle et al., 2011) can affect the way in which a face is perceived. For this study, we selected the subset of 93 photographs of White males with neutral expressions.

Using the Chicago Face Database supposes another advantage for our study. Each photograph is accompanied by information about the target face, and it has been rated by 74 participants on average (this number was calculated with the information available in Ma et al., 2015) on several facial traits: Afraid, Angry, Attractive, Baby-faced, Disgusted, Dominant, Feminine, Happy, Masculine, Prototypic, Sad, Surprised, Threatening, Trustworthy, and Unusual. Participants responded on a 1 to 7 Likert-type scale (1 = *not at all*, 7 = *extremely*) except for Prototypic, that was responded to on a 1 to 5 Likert-type scale. Prototypic was defined as to which degree the face seems typical. The raters showed a high degree of agreement in their assessments of the targets in all the traits. Detailed information on the database generation and characteristics of the participants is available in Ma et al. (2015). The mean scores of each facial trait for each face in our subset of 93 photographs are shown in Table S1 in Supplementary Material.

The facial features analyzed in this work were selected considering previous studies. Internal features (i.e., eyes, nose and mouth) seem to have significant importance in face recognition (Keil, 2009; Kwart et al., 2012). Among the internal features, eyes play a key role in face information processing (Fox & Damjanovic, 2006). Some authors include the eyebrows in the eye area (Saavedra et al., 2013; Sadr et al., 2003) or consider the eyebrows as a major factor in the perception of a face (Lundqvist et al., 1999). Blais et al. (2012) found that the mouth area is an important cue for both static and dynamic facial expressions, which was consistent with previous researches (Terry, 1977). However, external facial features such as hair or the shapes of the cheek, the chin, or the jaw also play an important role in the way in which the brain process the face information. According to Axelrod and Yovel (2010), the fusiform face area of the brain is not only sensitive to external features but also sensitive to their influence on the representation of internal facial features. Some works found that the face shape contributes significantly to face discrimination (Logan et al., 2017; Yamaguchi et al., 2013). From these previous works, we decided to consider the internal facial features (eyebrows, eyes, nose, and mouth) and the jaw contour in this study. Although other features have effects on face perception, for example, hair and facial hair, skin tone, and facial proportions (Dixson et al., 2016; Fink et al., 2006; Hagiwara et al., 2012; Jones et al., 2004; Pallett et al., 2010; Tsankova & Kappas, 2015), we limited our study to those features that have a main effect on face perception, rather than considering features that may vary from time to time such as hair (people can get a haircut).

### Classification of Facial Features by Appearance

To classify a big set of facial features is a complex task for humans. Classifying by appearance a very big set of elements in an undefined number of groups easily overwhelms our capacities for information processing (Miller, 1956; Scharff et al., 2011), and algorithms are more consistent for this task. Moreover, our brain integrates facial features into a gestalt whole when it processes a face’s information (Tanaka & Farah, 1993; Young et al., 1987), decreasing our ability for processing individual traits or parts of faces (Taubert et al., 2011). On the other hand, individual differences exist in face recognition ability (Wang et al., 2012) and some matters, like the race of the face, influence the performance in processing features and the configuration of facial information (Hayward et al., 2008; Rhodes et al., 2009). This is reflected in low interobserver and intraobserver agreement in the evaluation of facial features (Ritz-Timme et al., 2011). To deal with these problems, we propose an automatic procedure to perform this task.

**Figure 1.**
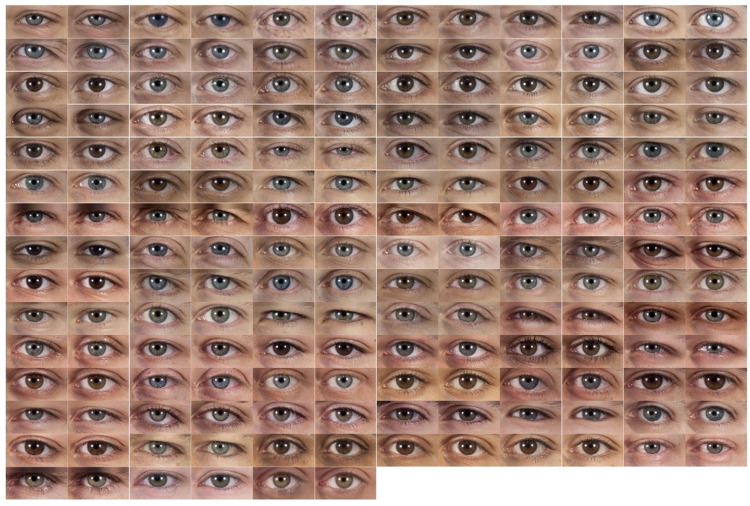
Left and Right Eye Images Obtained From 93 Photographs of White Males With Neutral Expressions. Right eyes were horizontally mirrored.

In a previous work (Fuentes-Hurtado et al., 2019), we developed an algorithm to automatically process images from the database and to extract individual images of the facial features of each face. Our objective was to extract the internal features (eyebrows, eyes, nose, and mouth) and the jaw contour. The RGB full-face photographs were used as the input of the algorithm for facial features extraction. The facial landmarks of each feature were detected, and the features separately extracted in images of the same size for each feature. This was accomplished using the CHEHRA facial key-point detector (Asthana et al., 2014). In this way, a set of landmarks was obtained for each photograph and, based on these landmarks, a mask for each feature was automatically created. The masks were used to extract the part of the image corresponding to each facial feature. The features were aligned with respect to the centroid of the previously acquired landmarks and saved as individual files. Figure 1 shows the set of eyes obtained using this procedure on our set of 93 photographs of White males with neutral expressions.

**Figure 2.**
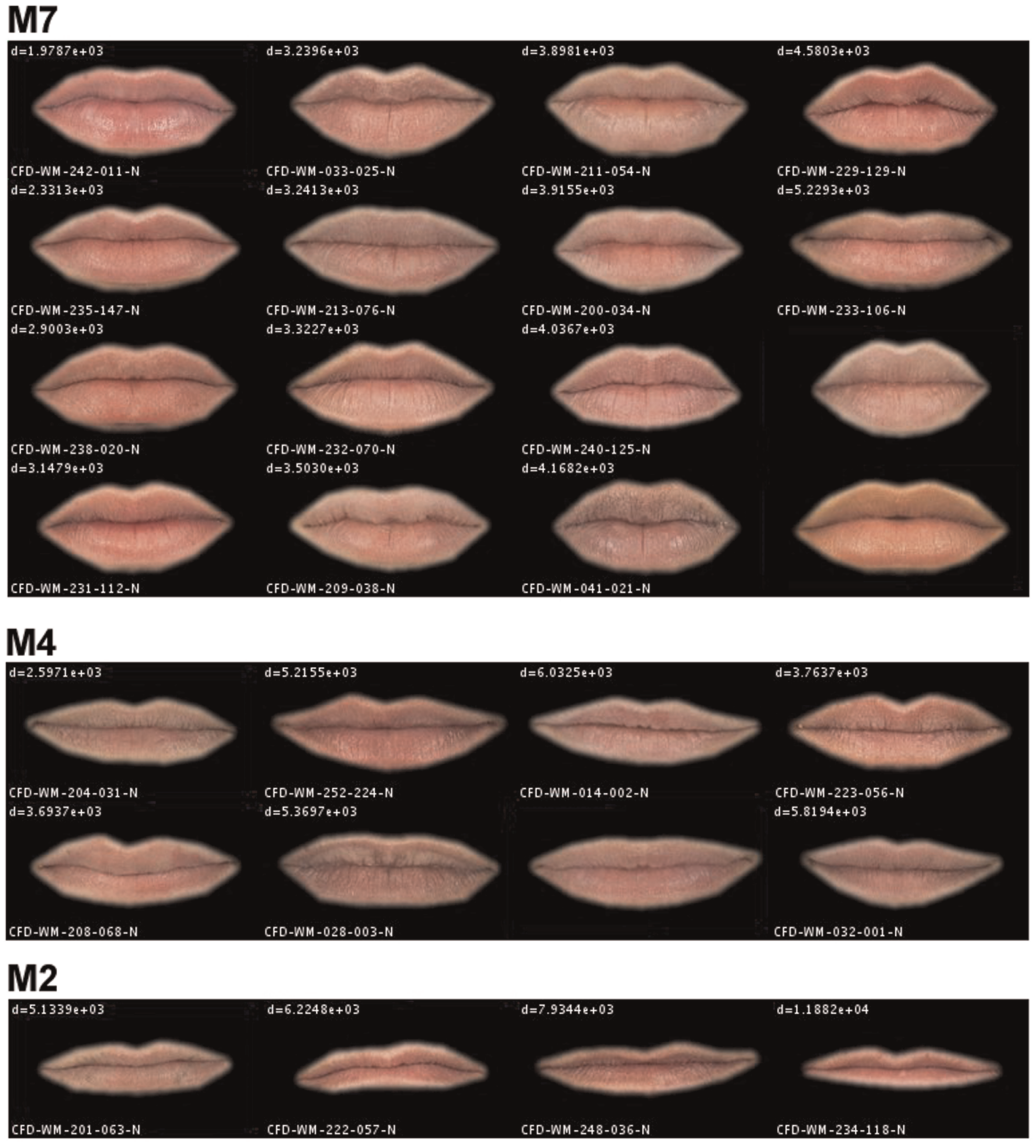
Members of the Clusters M7, M4 and M2 of Mouths.

Figure 2 shows the set of mouths extracted from the database. As can be seen, the background of the images of the mouths is black. This is because the presence of hair around the mouth of men is common. In our first tests, we detected that the presence of hair greatly affected the process of grouping the mouths; therefore, we decided to remove the surroundings of the original mouth obtaining a *shaved* mouth. The procedure followed to *shave* the mouths was as follows: First, the outer landmarks of the mouth were selected to form a polygon. Then, this polygon was enlarged by five pixels in every direction to ensure the whole mouth was taken inside the mask. Finally, a Gaussian Blur Filter (sigma = two pixels) was applied to the mask in order to smooth the transition between the skin and the black background of the image.

At this point, PCA (Sirovich & Kirby, 1987) was used on the five sets of images (one per feature type) in order to characterize each facial feature. Before proceeding, all the obtained images were converted to gray scale. In mathematical terms, this PCA aims to find the principal components of the distribution of faces, or the eigenvectors of the covariance matrix of the set of face images, treating each image as a vector in a very high dimensional space. These eigenvectors can be thought of as a set of features that together characterize the variation between images and are ordered accounting for the explained variance. Each individual face can be represented exactly in terms of a linear combination of the eigenvectors, or using the eigenvectors that explain the largest variances, and therefore account for the most variation within the set of images. The first M eigenvectors span an M-dimensional subspace of all possible images. Using this procedure over each set of facial features, it was possible to characterize each feature by a set of M eigenvalues, reducing the quantity of information used to describe the features. This procedure allows us to consider the global appearance of facial features while summarizing the central information to characterize them.

A PCA was performed on each subset of facial features. To facilitate the subsequent clustering process, the same number of eigenvectors (45) for each subset was selected bearing in mind that the explained variances were about 85% or higher in all cases and including more eigenvectors caused negligible gains (Fuentes-Hurtado et al., 2019). At this stage, the appearance of each feature could be characterized using 45 real values (eigenvalues). A K-means clustering algorithm (Macqueen, 1967) was selected to cluster the facial features using their eigenvalues as characteristics. A drawback of using this method is that the number of clusters (K) must be predefined. The approach used to face this problem was to perform several K-means executions varying K and to calculate the Dunn’s Index (Dunn, 1974) for each set of clusters. The Dunn’s Index measures the compactness and separation of the clusters obtained for each K. A higher Dunn’s Index points to a small intracluster variance and a high intercluster distance, namely, that the features included in each cluster are more similar to each other, and more different from the features belonging to other clusters. Therefore, we carried out several executions of the K-means algorithm varying K between 5 and 25, and the number of clusters for each feature was selected as the K that maximized the Dunn’s Index. The detailed clustering procedure and the complete set of results can be found in Fuentes-Hurtado (2018) and Fuentes-Hurtado et al. (2019).

Using this procedure, eyebrows were classified in 10 clusters (EB1 to EB10), eyes in 16 (E1 to E16), noses in 12 clusters (N1 to N12), mouths in 9 clusters (M1 to M9), and jaws in 11 (J1 to J11). As an example, Figure 2 shows the mouths included in the clusters M7, M4, and M2. Each facial feature in this figure is coded with the name of the face to which it belongs. Therefore, it is possible to classify each one of the 93 faces of the database using the clusters of its features. The last five columns of Table S1 in Supplementary Materials show the clusters of the facial features of the 93 faces of the database.

### Design

A general linear model (Searle, 1983) was fitted for each facial trait. Each model had five fixed factors (eyebrow, eye, nose, mouth, and jaw) and one of the 15 facial traits as dependent variable. The values of the dependent variables were the average ratings provided by human subjects (Ma et al., 2015). Our available data set consists of 93 observations with missing factors combinations. The 15 models were built considering only the main effects, without interactions. Some observations with abnormal studentized residuals were considered outliers and removed from the models. Depending on the facial trait analyzed, between zero and six faces were removed from the sample (marked with an asterisk in Table S1 in Supplementary Materials). The data were analyzed for each facial trait using an analysis of variance (ANOVA) with eyebrow (EB1–EB10), eye (E1–E16), nose (N1–N12), mouth (M1–M9), and jaw (J1–J11) as the main factors. The IBM SPSS Statistics 23.0 and Statgraphics Centurion v.XVII.II programs were used. The significance level was set at .05 for all tests.

## Results

The detailed results of each analysis for the effect of facial features on facial traits can be consulted in Table S3 in Supplementary Materials. The overall ANOVA results were statistically significant for all facial traits except Disgusted, *F*(53, 39) = 1.163, *p* = .313. It is not possible to reject the null hypothesis that all the data come from groups that have identical means for Disgusted. Because of this, and as no significant effects of facial features were found on this facial trait, Disgusted will be excluded from the results and conclusions detailed hereafter. For the remaining 14 traits, *R*^2^ varied in the range of .738 (Happy) to .898 (Prototypic), with adjusted *R*^2^ values of .340 and .744, respectively. The fitted models (predicted vs. observed values) are shown in Figure S1 in Supplementary Materials. Individual tests found different statistically significant main effects by trait (Table 1 shows the significant effects with bold characters). The effect size of each facial feature on the dependent variable was measured by means of partial eta squared (η_p_^2^). In this case, η_p_^2^ describes the proportion of total variance in each facial trait attributable to each facial feature. The complete set of values of η_p_^2^ is shown in the ANOVA tables in Supplementary Materials (Table S3), and the effect size of each facial feature by facial trait is shown in Table 1.

**Table 1.** Effect Size (η_p_^2^) and Statistical Significance of the Effects of Facial Features on Facial Traits.

	Eyebrow	Eye	Nose	Mouth	Jaw
Afraid					
Effect size	**η_p_^2^ = 0.42**	**η_p_^2^ = 0.70**	**η_p_^2^ = 0.59**	**η_p_^2^ = 0.51**	**η_p_^2^ = 0.55**
Significance	***F*(9,34) = 2.717** ***p* = .017**	***F*(14,34) = 5.675** ***p* < .0001**	***F*(11,34) = 4.520** ***p* < .0001**	***F*(8,34) = 4.375** ***p* = .001**	***F*(10,34) = 4.144** ***p* = .001**
Angry					
Effect size	**η_p_^2^ = 0.36**	**η_p_^2^ = 0.62**	η_p_^2^ = 0.24	**η_p_^2^ = 0.53**	**η_p_^2^ = 0.54**
Significance	***F*(9,35) = 2.182** ***p* = .048**	***F*(15,35) = 3.779** ***p* = .001**	*F*(11,35) = 0.988 *p* = .475	***F*(8,35) = 4.994** ***p* < .0001**	***F*(10,35) = 4.102** ***p* = .001**
Attractive					
Effect size	**η_p_^2^ = 0.52**	**η_p_^2^ = 0.62**	**η_p_^2^ = 0.53**	**η_p_^2^ = 0.48**	**η_p_^2^ = 0.49**
Significance	***F*(9,34) = 4.140** ***p* = .001**	***F*(14,34) = 3.918** ***p* = .001**	***F*(11,34) = 3.484** ***p* = .002**	***F*(1834) = 3.842** ***p* = .003**	***F*(10,34) = 3.288** ***p* = .005**
Baby-faced					
Effect size	η_p_^2^ = 0.29	**η_p_^2^ = 0.54**	η_p_^2^ = 0.37	**η_p_^2^ = 0.34**	**η_p_^2^ = 0.37**
Significance	*F*(9,36) = 1.631 *p* = .143	***F*(15,36) = 2.816** ***p* = .006**	*F*(11,36) = 1.892 *p* = .074	***F*(8,36) = 2.358** ***p* = .038**	***F*(10,36) = 2.132** ***p* = .047**
Disgusted					
Effect size	η_p_^2^ = 0.19	η_p_^2^ = 0.41	η_p_^2^ = 0.17	η_p_^2^ = 0.24	η_p_^2^ = 0.29
Significance	*F*(9,39) = 1.028 *p* = .435	*F*(15,39) = 1.782 *p* = .074	*F*(11,39) = 0.689 *p* = .732	*F*(8,39) = 1.517 *p* = .183	*F*(1,39) = 1.576 *p* = .150
Dominant					
Effect size	**η_p_^2^ = 0.42**	η_p_^2^ = 0.42	**η_p_^2^ = 0.43**	η_p_^2^ = 0.33	**η_p_^2^ = 0.42**
Significance	***F*(9,34) = 2.704** ***p* = .017**	*F*(14,34) = 1.784 *p* = .084	***F*(11,34) = 2.364** ***p* = .027**	*F*(8,34) = 2.064 *p* = .068	***F*(01,34) = 2.409** ***p* = .027**
Feminine					
Effect size	η_p_^2^ = 0.24	η_p_^2^ = 0.44	**η_p_^2^ = 0.43**	**η_p_^2^ = 0.51**	**η_p_^2^ = 0.43**
Significance	*F*(9,34) = 1.189 *p* = .333	*F*(15,34) = 1.788 *p* = .079	***F*(11,34) = 2.354** ***p* = .028**	***F*(8,34) = 4.336** ***p* = .001**	***F*(10,34) = 2.560** ***p* = .020**
Happy					
Effect size	η_p_^2^ = 0.26	η_p_^2^ = 0.24	η_p_^2^ = 0.17	**η_p_^2^ = 0.47**	**η_p_^2^ = 0.44**
Significance	*F*(9,35) = 1.381 *p* = .234	*F*(15,35) = 0.716 *p* = .752	*F*(11,35) = 0.644 *p* = .779	***F*(8,35) = 3.912** ***p* = .002**	***F*(10,35) = 2.734** ***p* = .013**
Masculine					
Effect size	η_p_^2^ = 0.33	**η_p_^2^ = 0.64**	η_p_^2^ = 0.27	**η_p_^2^ = 0.35**	η_p_^2^ = 0.38
Significance	*F*(9, 34) = 1.840 *p* = .097	***F*(15,34) = 3.997** ***p* < .0001**	*F*(11, 34) =1.162 *p* = .348	***F*(8,34) = 2.307** ***p* = .043**	*F*(10,34) =2.087 *p* = .054
Prototypic					
Effect size	**η_p_^2^ = 0.62**	**η_p_^2^ = 0.70**	η_p_^2^ = 0.39	**η_p_^2^ = 0.39**	**η_p_^2^ = 0.43**
Significance	***F*(9,35) = 6.297** ***p* < .0001**	***F*(15,35) = 5.313** ***p* < .0001**	*F*(11,35) = 2.043 *p* = .054	***F*(8,35) = 2.848** ***p* = .015**	***F*(10,35) = 2.621** ***p* = .017**
Sad					
Effect size	**η_p_^2^ = 0.39**	**η_p_^2^ = 0.49**	**η_p_^2^ = 0.46**	**η_p_^2^ = 0.39**	**η_p_^2^ = 0.44**
Significance	***F*(9,36) = 2.576** ***p* = .021**	***F*(15,36) = 2.289** ***p* = .021**	***F*(11,36) = 2.827** ***p* = .009**	***F*(8,36) = 2.828** ***p* = .015**	***F*(10,36) = 2.787** ***p* = .012**
Surprised					
Effect size	**η_p_^2^ = 0.37**	**η_p_^2^ = 0.45**	**η_p_^2^ = 0.41**	η_p_^2^ = 0.31	**η_p_^2^ = 0.45**
Significance	***F*(9,38) = 2.476** ***p* = .025**	***F*(15,38) = 2.060** ***p* = .036**	***F*(11,38) = 2.350** ***p* = .025**	*F*(8,38) = 2.141 *p* = .055	***F*(10,38) = 3.062** ***p* = .006**
Threatening					
Effect size	**η_p_^2^ = 0.52**	**η_p_^2^ = 0.57**	η_p_^2^ = 0.38	**η_p_^2^ = 0.39**	**η_p_^2^ = 0.52**
Significance	***F*(9,36) = 4.263** ***p* = .001**	***F*(14,36) = 3.393** ***p* = .002**	*F*(11,36) = 2.010 *p* = .057	***F*(8,36) = 2.821** ***p* = .015**	***F*(10,36) = 3.822** ***p* = .001**
Trustworthy					
Effect size	**η_p_^2^ = 0.54**	**η_p_^2^ = 0.60**	**η_p_^2^ = 0.51**	**η_p_^2^ = 0.49**	**η_p_^2^ = 0.45**
Significance	***F*(9,34) = 4.436** ***p* = .001**	***F*(15,34) = 3.358** ***p* = .002**	***F*(11,34) = 3.270** ***p* = .004**	***F*(8,34) = 4.033** ***p* = .002**	***F*(10,34) = 2.742** ***p* = .014**
Unusual					
Effect size	**η_p_^2^ = 0.39**	**η_p_^2^ = 0.57**	**η_p_^2^ = 0.41**	η_p_^2^ = 0.33	**η_p_^2^ = 0.40**
Significance	***F*(9,36) = 2.553** ***p* = .022**	***F*(15,36) = 3.142** ***p* = .002**	***F*(11,36) = 2.270** ***p* = .032**	*F*(8,36) = 2.162 *p* = .055	***F*(10,36) = 2.406** ***p* = .026**

*Note.* Significant effects are in boldface.

**Figure 3.**
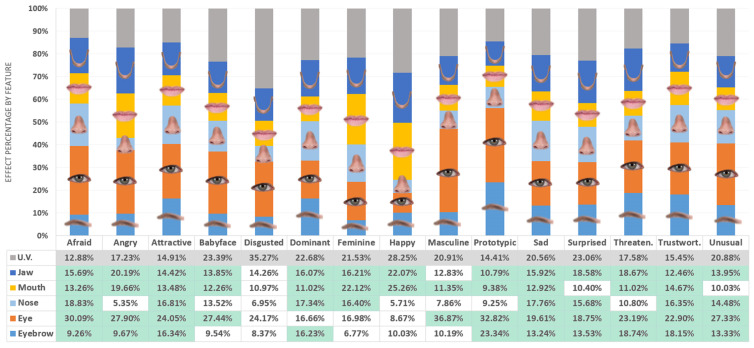
Effect Size of Each Facial Feature on Observed Facial Traits as a Percentage of All the Effects. Grayed area of bars and grayed cells represent variance unaccounted by the model (U.V.). Significant effects in green cells.

η_p_^2^ is usually preferred for effect size measurements in ANOVA (Richardson, 2011). However, to represent the effects size as percentages, another statistic, eta squared (η^2^), can be used for effect size measurements (Levine & Hullett, 2002). η^2^ was calculated for each facial feature as the sums of squares for the feature divided by the total sums of squares for all effects and errors in the corresponding ANOVA study. Figure 3 shows the effect size of each facial feature on each facial trait as a percentage of all the effects, including the effect of the model error. Figure S2 in Supplementary Materials shows the effects on each facial trait by facial feature.

To check if the assumptions for ANOVA were met, the residuals of each adjusted model were tested for normality and heteroscedasticity. We assumed that each observation was independent from all other observations. Each face in the sample belongs to a different person randomly selected. In Figure S1 in Supplementary Materials, the scatterplots of residuals on predicted values of each model suggest that errors have constant variance, with the residuals scattered randomly around zero. The plots of normal probability of the residuals (Figure S1) suggest evidence of normality. Supplementary Kolmogorov–Smirnov tests were performed and skewness and kurtosis checked (α = .05). The results in the Table S2 show that null hypothesis cannot be rejected and conclude normality is a reasonable assumption for the residuals of all the facial traits.

## Discussion

The main objective of this work was to measure how much the appearance of the facial features affects the opinion of the observers about some facial traits. We have proposed a new approach to relate 5 facial features of White males with 15 perceived facial traits. First, we selected 93 faces of White males with 15 facial traits assessed by human observers. Then, the facial features of the faces were categorized by appearance. To achieve this, we used an automatic procedure to extract the facial features from the whole face images, and PCA and K-means clustering algorithm were applied to characterize and group the facial features by their global appearance. Features belonging to the same category or cluster have a similar appearance, and it is supposed to have the same effect size on the face traits. In this way, our sample of 93 whole faces were classified by the clusters to which their features belong. Finally, general linear models relating the facial features of the faces to the observed facial traits were fitted for each trait. The effect size of each facial feature on the perceived facial traits was measured using the partial eta squared.

Classic behavioral work has shown that the human brain integrates facial features into a gestalt whole when it processes facial information (Tanaka & Farah, 1993; Young et al., 1987), diminishing our capacity for processing facial information of faces with missing features or parts (Taubert et al., 2011). Therefore, information from all the facial features is used and plays a significant role in judging facial traits. Globally, our results are compatible with this, showing that most of the facial features have a statistically significant effect on how an observer perceives facial traits. In accordance with Cohen (1988), all of them can be considered large effects (η_p_^2^ > 0.1379).

However, the size of the effect varies for each facial feature. Our results show that eyes is the facial feature whose appearance has the biggest effect on perceived facial traits. The average percentage of effect on all the facial traits is 23.83%. Particularly, the effect of the eyes is over 30% on Masculine, Prototypic, and Afraid, being under 15% only on Happy (8.67%). The appearance of the jaw has the second mean effect size (15.73%), being the most homogenous among the different traits analyzed. This result is consistent with previous studies that found that face shape contributes significantly to face processing (Logan et al., 2017; Yamaguchi et al., 2013). The mouth has a big effect on Happy, Feminine, and Angry (over 19%). This feature is related with dynamic facial expressions (Blais et al., 2012) and expresses emotions of happiness (e.g., smiling or laughing) and anger (e.g., screaming or shouting out) changing its shape. Therefore, the obtained results seems to be consistent with preceding works that found that people make trait inferences driven by structural resemblance to emotional expressions, even in neutral faces (Petrican et al., 2014; Said et al., 2009). On the other hand, our results show that jaw also has great effects on Happy and Angry. In the same way as in the case of the mouth, this could be related to the changes in the shape of the jaw when the face expresses happiness or anger, but this must be studied further.

The appearance of the eyebrows mean effect size is 13.11% being its most significant effect on Prototypic (23.34%). Therefore, eyebrows are an important clue to decide to which degree a face is the typical face of a White man. Lundqvist et al. (1999) found that the eyebrows are the most important feature for conveying facial threat. In the same way, our results show that eyebrows plays an important role in Threatening (18.74%). However, we found that eyes has more effect on Threatening (23.19%) than eyebrows. The difference in the results for the eyes may be due to the fact that they used schematic faces in their study and we have employed real faces. Nose has the smallest average effect (12.87%) on perceived facial traits. Its major effects are on Afraid, Sad, Dominant, Attractive, Feminine, and Trustworthy and are almost negligible on Happy and Angry.

In this study, η^2^ and η_p_^2^ have been used to measure the effect size of each facial feature on each social trait. We have used both because η^2^ is more intuitive to measure the effect sizes of the variables than η_p_^2^, but the former makes it hard to compare the effect of a single variable in different studies, and the proportion explained by any one variable depends on the number of variables on the model.

The obtained models were statistically significant (except Disgusted model) with *R*^2^ varying in the range of .738 to .898. Therefore, most of the variation in each facial trait can be explained by the different class of the analyzed facial features. However, unexplained variation remains. Our results show higher unexplained variance in the models for Happy, Baby-faced, Surprised, or Dominant than those models for Attractive, Prototypic, or Afraid. It can be argued that the amount of unexplained variation in each model depends on the differences between the processes to assess each facial trait and the capacity of the models to capture the inherent variance of each kind of assessment. Ours models only consider the main effect of each independent variable, which may affect the capacity of the models to capture the variance to a different extent for each facial trait. On the other hand, faces with neutral expressions were assessed to create the models. This can lead to more unexplained variance in the models of the facial traits more related to facial expressions. For example, the differences between neutral and happy faces are larger than that between neutral and fearful faces (Leppänen & Hietanen, 2007), making it more difficult to assess the happy versus fearful trait of a neutral face. Finally, our procedure does not consider the whole face space. Each facial trait may be linked to facial features not included in this study (such as hair or skin tone) to a different degree, leading to differences in the amount of unexplained variance between models.

As far as we know, this is the most comprehensive work, in terms of number of traits considered, measuring the effect size of the appearance of the facial features on the perception of facial traits. Other techniques, such us bubbles (Gosselin & Schyns, 2001) or reverse correlation methods (Dotsch & Todorov, 2012; Todorov et al., 2011), can discriminate the features used by observers in categorization tasks. Our technique differs from these in several aspects. Our approach rests on the assumption that the global appearance of the facial features can be a good predictor of how features are used to assess the traits of the face. If the clustering procedure achieves enough intraclass homogeneity and interclass heterogeneity, facial features belonging to the same class (with similar overall appearance) will lead to similar assessments, and these judgments will be dissimilar from those of faces with facial features belonging to other clusters. Our models use five meaningful independent variables each one describing a complete facial feature, rather than a collection of smaller features that conform faces (lines, shadows, surfaces . . . ) or disconnected areas of the faces. Therefore, a more direct link can be established between perceived facial traits and what people intuitively consider an eye, an eyebrow, a nose, a mouth, and a jaw.

On the other hand, our technique allows judgments to be made on complete real faces. Some other approaches involve using graphically manipulated photographs in the categorization tasks, by superimposing noise or hiding parts of the faces. In these approaches, judgments are made on synthetic, noisy, or partial faces that can influence the assessment.

However, some limitations of this study must be pointed out, mainly regarding the interactions between the facial features. In this work, we are measuring the main effect sizes. The main effect of each feature can explain part of the variation within the face appraisals (Cabeza & Kato, 2000; Rakover, 2002). To consider the second-order effects (the effect of one feature’s appearance considering the other features’ appearance), a larger sample of images of real faces is needed. However, it is very difficult to develop a big enough face database and to collect ratings for all the faces. Moreover, it is not possible to find real faces with all the possible combinations of facial features. A usual way to achieve this is to collect ratings for faces in which specific areas of the face are parametrically manipulated in terms of size or shape. However, changing some relevant dimension of the facial features is not appropriate for this case because the classifications of facial features are based on their overall appearance.

On the other hand, we have followed a mixed feature-based/image-based approach to obtain the effect sizes of the appearance of the features. We have established a reduced set of relevant specific attributes to describe a face (five basic facial features) and used an image-based approach (PCA) to categorize these facial features by their global appearance. Therefore, although the faces were described by five meaningful variables, the configural information of the faces is not explicitly considered to obtain the effect size of the facial features.

Regarding the generalization of the findings, 93 faces of the Chicago Face Database were used to obtain the models relating facial features to facial trait assessments. The faces of the database belong to White men between the ages of 18 and 40 living in the Chicago (U.S.A) area. The subjective classifications of the faces were made by a specific group of women and men probably from the same city (Ma et al., 2015). Therefore, both the faces and the appraisals used to develop the models come from a specific community. The generalization of the results to faces of people from other communities must be carefully addressed. Especially, the findings cannot be generalized to faces of people of other races (Hayward et al., 2008; Rhodes et al., 2009) or to faces of people outside the range of this study.

Our future works will try to develop similar studies for female faces and to extend the results to other races. On the other hand, using a larger face database would allow us to consider interactions, at least of the second order, among the facial features, or to include more facial features in the models.

## Supplemental Material

sj-pdf-1-ipe-10.1177_2041669520961123 - Supplemental material for The Influence of Each Facial Feature on How We Perceive and Interpret Human Faces Click here for additional data file. Supplemental material, sj-pdf-1-ipe-10.1177_2041669520961123 for The Influence of Each Facial Feature on How We Perceive and Interpret Human Faces by Jose A. Diego-Mas, Felix Fuentes-Hurtado, Valery Naranjo and Mariano Alcañiz in i-Perception

## References

[bibr1-2041669520961123] Ahonen T. Hadid A. Pietikäinen M. (2006). Face description with local binary patterns: Application to face recognition. IEEE Transactions on Pattern Analysis and Machine Intelligence, 28(12), 2037–2041. 10.1109/TPAMI.2006.244 17108377

[bibr2-2041669520961123] Åsli O. Michalsen H. Øvervoll M. (2017). In your face: Startle to emotional facial expressions depends on face direction. i-Perception, *8*(1), 1--13. 10.1177/2041669517694396 PMC5347266 28321290

[bibr3-2041669520961123] Asthana A. Zafeiriou S. Cheng S. Pantic M. (2014). Incremental face alignment in the wild. In *Proceedings of the IEEE computer society conference on computer vision and pattern recognition*, pp. 1859–1866. Columbus, OH, USA: IEEE. 10.1109/CVPR.2014.240

[bibr4-2041669520961123] Axelrod V. Yovel G. (2010). External facial features modify the representation of internal facial features in the fusiform face area. NeuroImage, 52(2), 720–725. 10.1016/j.neuroimage.2010.04.027 20406694

[bibr5-2041669520961123] Bartlett, J. C., Searcy, J. H., & Abdi, H. (2003). What are the routes to face recognition? In M. A. Peterson & G. Rhodes (Eds.), *Advances in visual cognition. Perception of faces, objects, and scenes: Analytic and holistic processes* (pp. 21--47). Oxford University Press. https://doi.org/10.1093/acprof:oso/9780195313659.003.0002

[bibr6-2041669520961123] Belhumeur P. N. Hespanha J. P. Kriegman D. J. (1997). Eigenfaces vs. fisherfaces: Recognition using class specific linear projection. IEEE Transactions on Pattern Analysis and Machine Intelligence, 19(7), 711–720. 10.1109/34.598228

[bibr7-2041669520961123] Biederman I. (1987). Recognition by components: A theory of human image understanding. Psychological Review, 94(2), 115–117. 10.1037/0033-295X.94.2.115 3575582

[bibr8-2041669520961123] Blais C. Roy C. Fiset D. Arguin M. Gosselin F. (2012). The eyes are not the window to basic emotions. Neuropsychologia, 50(12), 2830–2838. 10.1016/j.neuropsychologia.2012.08.010 22974675

[bibr9-2041669520961123] Bovet J. Barthes J. Durand V. Raymond M. Alvergne A. (2012). Men’s preference for women’s facial features: Testing homogamy and the paternity uncertainty hypothesis. PLoS One, 7(11), 1--11. 10.1371/journal.pone.0049791 PMC3504097 23185437

[bibr10-2041669520961123] Brahnam S. Nanni L. (2010). Predicting trait impressions of faces using local face recognition techniques. Expert Systems With Applications, 37(7), 5086–5093. 10.1016/j.eswa.2009.12.002

[bibr11-2041669520961123] Bruce V. Young A. (1986). Understanding face recognition. British Journal of Psychology, 77(Pt 3), 305–327. 10.1111/j.2044-8295.1986.tb02199.x 3756376

[bibr12-2041669520961123] Bruce V. Young A. (2012). Face perception. Psychology Press.

[bibr13-2041669520961123] Cabeza R. Kato T. (2000). Features are also important: Contributions of featural and configural processing to face recognition. Psychological Science, 11(5), 429–433. 10.1111/1467-9280.00283 11228917

[bibr14-2041669520961123] Chihaoui M. Elkefi A. Bellil W. Ben Amar C. (2016). A survey of 2D face recognition techniques. Computers, 5(4), 21 10.3390/computers5040021

[bibr15-2041669520961123] Cohen, J. (1988). *Statistical Power Analysis for the Behavioral Sciences* (2nd ed. p. 567). Hillsdale, NJ: Lawrence Erlbaum Associates, Publishers.

[bibr16-2041669520961123] Cootes T. F. Edwards G. J. Taylor C. J. (2001). Active appearance models. IEEE Transactions on Pattern Analysis and Machine Intelligence, 23(6), 681–685. 10.1007/BFb0054760

[bibr17-2041669520961123] Damasio, A. R. (1985). Prosopagnosia. *Trends in Neuroscience, 8*(1), 132--135.

[bibr18-2041669520961123] Diamond R. Carey S. (1986). Why faces are and are not special. An effect of expertise. Journal of Experimental Psychology: General, 115(2), 107–117. 10.1037/0096-3445.115.2.107 2940312

[bibr19-2041669520961123] Dixson B. J. W. Sulikowski D. Gouda-Vossos A. Rantala M. J. Brooks R. C. (2016). The masculinity paradox: Facial masculinity and beardedness interact to determine women’s ratings of men’s facial attractiveness. Journal of Evolutionary Biology, 29(11), 2311–2320. 10.1111/jeb.12958 27488414

[bibr20-2041669520961123] Dizaji K. G. Herandi A. Deng C. Cai W. Huang H. (2017). Deep clustering via joint convolutional autoencoder embedding and relative entropy minimization. In: *Proceedings of the IEEE international conference on computer vision* IEEE, pp. 5747--5756. 10.1109/ICCV.2017.612

[bibr21-2041669520961123] Dotsch, R., & Todorov, A. (2012). Reverse Correlating Social Face Perception. *Social Psychological and Personality Science, 3*(5), 562--571. 10.1177/1948550611430272

[bibr22-2041669520961123] Dunn J. C. (1974). Well-separated clusters and optimal fuzzy partitions. Journal of Cybernetics, 4(1), 95–104. 10.1080/01969727408546059

[bibr23-2041669520961123] Eberhardt J. L. Davies P. G. Purdie-Vaughns V. J. Johnson S. L. (2006). Looking deathworthy perceived stereotypicality of black defendants predicts capital-sentencing outcomes. Psychological Science, 17(5), 383–386. 10.1111/j.1467-9280.2006.01716.x 16683924

[bibr24-2041669520961123] Favelle, S. K., Palmisano, S., & Avery, G. (2011). Face Viewpoint Effects about Three Axes: The Role of Configural and Featural Processing. *Perception, 40*(7), 761--784. 10.1068/p6878 22128550

[bibr25-2041669520961123] Fink B. Neave N. Manning J. T. Grammer K. (2006). Facial symmetry and judgements of attractiveness, health and personality. Personality and Individual Differences, 41(3), 491–499. 10.1016/j.paid.2006.01.017

[bibr26-2041669520961123] Fox E. Damjanovic L. (2006). The eyes are sufficient to produce a threat superiority effect. Emotion, 6(3), 534–539. 10.1037/1528-3542.6.3.534 16938095 PMC1852642

[bibr27-2041669520961123] Fuentes-Hurtado F. (2018). *A system for modeling social traits in realistic faces with artificial intelligence* [Unpublished master’s thesis]. Universidad Politécnica de Valencia. 10.4995/Thesis/10251/101943

[bibr28-2041669520961123] Fuentes-Hurtado F. Diego-Mas J. A. Naranjo V. Alcañiz M. (2019). Automatic classification of human facial features based on their appearance. PLoS One, 14(1), e0211314 10.1371/journal.pone.0211314 30695076 PMC6350975

[bibr29-2041669520961123] Gill D. (2017). Women and men integrate facial information differently in appraising the beauty of a face. Evolution and Human Behavior, 38(6), 756–760. 10.1016/j.evolhumbehav.2017.07.001

[bibr30-2041669520961123] Gosselin F. Schyns P. G. (2001). Bubbles: A technique to reveal the use of information in recognition tasks. Vision Research, 41(17), 2261–2271. 10.1016/S0042-6989(01)00097-9 11448718

[bibr31-2041669520961123] Hagiwara N. Kashy D. A. Cesario J. (2012). The independent effects of skin tone and facial features on Whites’ affective reactions to Blacks. Journal of Experimental Social Psychology, 48(4), 892–898. 10.1016/j.jesp.2012.02.001

[bibr32-2041669520961123] Hayward W. G. Rhodes G. Schwaninger A. (2008). An own-race advantage for components as well as configurations in face recognition. Cognition, 106(2), 1017--1027. 10.1016/j.cognition.2007.04.002 17524388

[bibr33-2041669520961123] Huang P. Huang Y. Wang W. Wang L. (2014). Deep embedding network for clustering. In *Proceedings—International conference on pattern recognition* (pp. 1532–1537). 10.1109/ICPR.2014.272

[bibr34-2041669520961123] Jack R. E. Schyns P. G. (2015). The human face as a dynamic tool for social communication. Current Biology, 25(14), R621–R634. 10.1016/J.CUB.2015.05.052 26196493

[bibr35-2041669520961123] Jones B. C. Little A. C. Burt D. M. Perrett D. I. (2004). When facial attractiveness is only skin deep. Perception, 33(5), 569–576. 10.1068/p3463 15250662

[bibr36-2041669520961123] Kanwisher N. McDermott J. Chun M. M. (1997). The fusiform face area: A module in human extrastriate cortex specialized for the perception of faces. Journal of Neuroscience, 17(11), 4302–4311. http://www.ncbi.nlm.nih.gov/pubmed/9151747 9151747 10.1523/JNEUROSCI.17-11-04302.1997 PMC6573547

[bibr37-2041669520961123] Keating C. F. Doyle J. (2002). The faces of desirable mates and dates contain mixed social status cues. Journal of Experimental Social Psychology, 38(4), 414–424. 10.1016/S0022-1031(02)00007-0

[bibr38-2041669520961123] Keil M. S. (2009). “I look in your eyes, honey”: Internal face features induce spatial frequency preference for human face processing. PLoS Computational Biology, 5(3), 1--13. 10.1371/journal.pcbi.1000329 PMC2653192 19325870

[bibr39-2041669520961123] Klare B. Jain A. K. (2010). On a taxonomy of facial features. In *IEEE 4th international conference on biometrics: Theory, applications and systems, BTAS 2010* (pp. 1–8). IEEE. 10.1109/BTAS.2010.5634533

[bibr40-2041669520961123] Kwart D. G. Foulsham T. Kingstone A. (2012). Age and beauty are in the eye of the beholder. Perception, 41(8), 925–938. 10.1068/p7136 23362670

[bibr41-2041669520961123] Langlois J. H. Kalakanis L. Rubenstein A. J. Larson A. Hallam M. Smoot M. (2000). Maxims or myths of beauty? A meta-analytic and theoretical review. Psychological Bulletin, 126(3), 390–423. 10.1037/0033-2909.126.3.390 10825783

[bibr42-2041669520961123] Leppˮen, J. M. & Hietanen, J. K. (2007) Is there more in a happy face than just a big smile?. *Visual Cognition, 15*(4), 468--490. https://doi.org/http://doi.org/10.1080/13506280600765333

[bibr43-2041669520961123] Levine T. R. Hullett C. R. (2002). Eta squared, partial eta squared, and misreporting of effect size in communication research. Human Communication Research, 28(4), 612–625. http://www.toposbooks.gr/behavioralstats/Levine_Hullett_2002.pdf

[bibr44-2041669520961123] Little A. C. Burriss R. P. Jones B. C. Roberts S. C. (2007). Facial appearance affects voting decisions. Evolution and Human Behavior, 28(1), 18–27. 10.1016/j.evolhumbehav.2006.09.002

[bibr45-2041669520961123] Logan A. J. Gordon G. E. Loffler G. (2017). Contributions of individual face features to face discrimination. Evolution and Human Behavior, 137, 29–39. 10.1016/j.visres.2017.05.011 28688904

[bibr46-2041669520961123] Lundqvist D. Esteves F. Öhman A. (1999). The face of wrath: Critical features for conveying facial threat. Cognition and Emotion, 13(6), 691–711. 10.1080/026999399379041 29148306

[bibr47-2041669520961123] Ma D. S. Correll J. Wittenbrink B. (2015). The Chicago face database: A free stimulus set of faces and norming data. Behavior Research Methods, 47(4), 1122–1135. 10.3758/s13428-014-0532-5 25582810

[bibr48-2041669520961123] Macqueen J. (1967). Some methods for classification and analysis of multivariate observations. Proceedings of the Fifth Berkeley Symposium on Mathematical Statistics and Probability, 1, 281–297. https://doi.org/citeulike-article-id:6083430

[bibr49-2041669520961123] Maloney L. T. Dal Martello M. F. (2006). Kin recognition and the perceived facial similarity of children. Journal of Vision, 6(10), 4 10.1167/6.10.4 17132076

[bibr50-2041669520961123] Mckone E. Yovel G. (2009). Why does picture-plane inversion sometimes dissociate perception of features and spacing in faces, and sometimes not? Toward a new theory of holistic processing. Psychonomic Bulletin and Review, 16(5), 778–797. 10.3758/PBR.16.5.778 19815781

[bibr51-2041669520961123] Meyers E. Wolf L. (2008). Using biologically inspired features for face processing. International Journal of Computer Vision, 76(1), 93–104. 10.1007/s11263-007-0058-8

[bibr52-2041669520961123] Miller G. (1956). The magical number seven, plus or minus two: Some limits on our capacity for processing information. Psychological Review, 101(2), 343–352. 10.1037/h0043158 8022966

[bibr53-2041669520961123] Pallett P. M. Link S. Lee K. (2010). New “golden” ratios for facial beauty. Vision Research, 50(2), 149–154. 10.1016/j.visres.2009.11.003 19896961 PMC2814183

[bibr54-2041669520961123] Paunonen S. V Ewan K. Earthy J. Lefave S. Goldberg H. (1999). Facial features as personality cues. Journal of Personality, 67(3), 555–583. 10.1111/1467-6494.00065

[bibr55-2041669520961123] Petrican R. Todorov A. Grady C. (2014). Personality at face value: Facial appearance predicts self and other personality judgments among strangers and spouses. Journal of Nonverbal Behavior, 38(2), 259–277. 10.1007/s10919-014-0175-3 27330234 PMC4909468

[bibr56-2041669520961123] Piepers D. W. Robbins R. A. (2012). A review and clarification of the terms “holistic”, “configural”, and “relational” in the face perception literature. Frontiers in Psychology, 3, 559 10.3389/fpsyg.2012.00559 23413184 PMC3571734

[bibr57-2041669520961123] Rakover S. S. (2002). Featural vs. configurational information in faces: A conceptual and empirical analysis. British Journal of Psychology, 93(1), 1–30. 10.1348/000712602162427 11839099

[bibr58-2041669520961123] Rhodes G. Ewing L. Hayward W. G. Maurer D. Mondloch C. J. Tanaka J. W. (2009). Contact and other-race effects in configural and component processing of faces. British Journal of Psychology, 100(4), 717–728. 10.1348/000712608X396503 19228441

[bibr59-2041669520961123] Richardson J. T. E. (2011). Eta squared and partial eta squared as measures of effect size in educational research. Educational Research Review, 6(2), 135–147. 10.1016/j.edurev.2010.12.001

[bibr60-2041669520961123] Ritz-Timme S. Gabriel P. Obertovà Z. Boguslawski M. Mayer F. Drabik A. Poppa P. De Angelis D. Ciaffi R. Zanotti B. Gibelli D. Cattaneo C. (2011). A new atlas for the evaluation of facial features: Advantages, limits, and applicability. International Journal of Legal Medicine, 125(2), 301–306. 10.1007/s00414-010-0446-4 20369248

[bibr61-2041669520961123] Rojas M. M. Masip D. Todorov A. Vitria J. (2011). Automatic prediction of facial trait judgments: Appearance vs. structural models. PLoS One, 6(8), 1--12. 10.1371/journal.pone.0023323 PMC3157350 21858069

[bibr62-2041669520961123] Rossion B. (2008). Picture-plane inversion leads to qualitative changes of face perception. Acta Psychologica, 128(2), 274–289. 10.1016/j.actpsy.2008.02.003 18396260

[bibr63-2041669520961123] Russell R. (2003). Sex, beauty, and the relative luminance of facial features. Perception, 32(9), 1093–1107. 10.1068/p5101 14651322

[bibr64-2041669520961123] Saavedra C. Smith P. Peissig J. (2013). The relative role of eyes, eyebrows, and eye region in face recognition. Journal of Vision, 13(9), 410 10.1167/13.9.410

[bibr65-2041669520961123] Sadr J. Jarudi I. Sinha P. (2003). The role of eyebrows in face recognition. Perception, 32(3), 285–293. 10.1068/p5027 12729380

[bibr66-2041669520961123] Said C. Sebe N. Todorov A. (2009). “Structural resemblance to emotional expressions predicts evaluation of emotionally neutral faces”: Correction to Said, Sebe, and Todorov (2009). Emotion, 9(4), 509–509. 10.1037/a0016784 19348537

[bibr67-2041669520961123] Scharff A. Palmer J. Moore C. M. (2011). Evidence of fixed capacity in visual object categorization. Psychonomic Bulletin & Review, 18(4), 713–721. 10.3758/s13423-011-0101-1 21538202 PMC6999811

[bibr68-2041669520961123] Schobert A.-K. Corradi-Dell’Acqua C. Frühholz S. van der Zwaag W. Vuilleumier P. (2018). Functional organization of face processing in the human superior temporal sulcus: A 7T high-resolution fMRI study. Social Cognitive and Affective Neuroscience, 13(1), 102--113. 10.1093/scan/nsx119 PMC5793830 29140527

[bibr69-2041669520961123] Searle, SR. (1983) General linear model. In: Kotz, S, Norman, LJ, Campbell, BR, eds. *Encyclopedia of Statistical Sciences*. New York: John Wiley & Sons Ltd, pp. 357--72.

[bibr70-2041669520961123] Sirovich L. Kirby M. (1987). Low-dimensional procedure for the characterization of human faces. Journal of the Optical Society of America. A, Optics and Image Science, 4(3), 519–524. 10.1364/JOSAA.4.000519 3572578

[bibr71-2041669520961123] Tanaka J. W. Farah M. J. (1993). Parts and wholes in face recognition. The Quarterly Journal of Experimental Psychology, 46(2), 225–245. 10.1080/14640749308401045 8316637

[bibr72-2041669520961123] Taubert J. Apthorp D. Aagten-Murphy D. Alais D. (2011). The role of holistic processing in face perception: Evidence from the face inversion effect. Vision Research, 51(11), 1273–1278. 10.1016/j.visres.2011.04.002 21496463

[bibr73-2041669520961123] Terry R. R. L. (1977). Further evidence on components of facial attractiveness. Perceptual and Motor Skills, 45(1), 130 10.2466/pms.1977.45.1.130

[bibr74-2041669520961123] Todorov, A. (2011). Evaluating faces on social dimensions. In A. Todorov, S. T. Fiske, & D. A. Prentice (Eds.), *Oxford series in social cognition and social neuroscience. Social neuroscience: Toward understanding the underpinnings of the social mind *(p. 54--76). Oxford University Press. https://doi.org/10.1093/acprof:oso/9780195316872.003.0004

[bibr75-2041669520961123] Todorov A. Dotsch R. Wigboldus D. H. J. Said C. P. (2011). Data-driven methods for modeling social perception. Social and Personality Psychology Compass, 5(10), 775–791. 10.1111/j.1751-9004.2011.00389.x

[bibr76-2041669520961123] Todorov A. Mandisodza A. N. Goren A. Hall C. C. (2005). Psychology: Inferences of competence from faces predict election outcomes. Science, 308(5728), 1623–1626. 10.1126/science.1110589 15947187

[bibr77-2041669520961123] Todorov A. Said C. P. Engell A. D. Oosterhof N. N. (2008). Understanding evaluation of faces on social dimensions. Trends in Cognitive Sciences, 12, 455–460. 10.1016/j.tics.2008.10.001 18951830

[bibr78-2041669520961123] Tsankova E. Kappas A. (2015). Facial skin smoothness as an indicator of perceived trustworthiness and related traits. Perception, 45(4), 400–408. 10.1177/0301006615616748 26621963

[bibr79-2041669520961123] Turk M. Pentland A. (1991). Eigenfaces for recognition. Journal of Cognitive Neuroscience, 3(1), 71–86. 10.1162/jocn.1991.3.1.71 23964806

[bibr80-2041669520961123] Wang R. Li J. Fang H. Tian M. Liu J. (2012). Individual differences in holistic processing predict face recognition ability. Psychological Science, 23(2), 169–177. 10.1177/0956797611420575 22222218

[bibr81-2041669520961123] Wilson J. P. Rule N. O. (2015). Facial trustworthiness predicts extreme criminal-sentencing outcomes. Psychological Science, 26(8), 1325–1331. 10.1177/0956797615590992 26162847

[bibr82-2041669520961123] Xie J. Girshick R. Farhadi A. (2016). Unsupervised deep embedding for clustering analysis. In (Balcan, M. F. & Weinberger, K.Q. eds) *Proceedings of the 33rd international conference on international conference on machine learning—Volume 48* (pp. 478–487). JMLR. https://dl.acm.org/citation.cfm?id=3045442

[bibr83-2041669520961123] Yamaguchi M. K. Hirukawa T. Kanazawa S. (2013). Judgment of gender through facial parts. Perception, 42(11), 1253–1265. 10.1068/p240563n 24601037

[bibr84-2041669520961123] Young, A. W., Hellawell, D., & Hay, D. C. (1987). Configurational information in face perception. *Perception, 16*(6), 747--759. 10.1068/p160747 3454432

[bibr816-2041669520961123] Zebrowitz, L. A., & Montepare, J. M. (2008). Social psychological face perception: Why appearance matters. *Social and Personality Psychology Compass, 2*(3), 1497--1517. https://doi.org/10.1111/j.1751-9004.2008.00109.x 10.1111/j.1751-9004.2008.00109.x PMC2811283 20107613

[bibr86-2041669520961123] Zebrowitz-McArthur L. Baron R. M. (1983). Toward an ecological theory of social perception. Psychological Review, 90(3), 215–238. 10.1037//0033-295X.90.3.215

