# Supplementary material for: The Influence of Each Facial Feature on How We Perceive and Interpret Human Faces
Source: Iperception. 2020 Sep 30;11(5):2041669520961123. doi: 10.1177/2041669520961123 (PMC7533946; doi:10.1177/2041669520961123)
Supplement: sj-pdf-1-ipe-10.1177_2041669520961123 - Supplemental material for The Influence of Each Facial Feature on How We Perceive and Interpret Human Faces [file sj-pdf-1-ipe-10.1177_2041669520961123.pdf]

The influence of each facial feature on how we perceive and interpret human faces

Supplementary materials

Jose A. Diego-Mas, Felix Fuentes-Hurtado, Valery Naranjo and Mariano Alcañiz

Table S1. Mean scores for each facial trait and classification of facial features for each face.

	Facial trait															Facial feature				
Face	Afraid	Angry	Attractive	Baby-faced	Disgusted	Dominant	Feminine	Happy	Masculine	Prototypic	Sad	Surprised	Threatening	Trustworthy	Unusual	Eyebrow	Eye	Nose	Mouth	Jaw
WM-001	2.548	2.247	2.645	3.591	2.183	1.857	2.097	1.968	3.581	3.857	3.495*	1.699	1.828	3.215	2.323	EB10	E9	N12	M8	J1
WM-002	2.038	3.570	2.513	1.788	3.025	3.857	1.696	2.013	4.875	1.873	2.775	1.788	3.525	2.975	3.313	EB3	E8	N2	M1	J10
WM-003	1.988	2.446	3.683	2.880	2.181	3.360	1.916	2.542	4.892	4.191	2.675	1.892	2.566	3.578	2.639	EB6	E16	N4	M4	J5
WM-004	1.804	1.835	4.663	2.467	1.739	3.154	1.804	2.630	4.848	4.288	2.435	1.467	1.761	3.560	1.934	EB8	E13	N2	M6	J10
WM-006	1.965	2.442	3.512	3.047	2.024	3.476	2.581*	2.753	4.477	3.857	2.440	1.756	2.477	3.430	2.929	EB6	E13	N2	M6	J9
WM-009	2.120	2.783	4.076	2.912	2.380	3.346	2.033	2.261	4.728	3.488	2.391	1.856	2.457	3.511	2.231	EB3	E11	N2	M6	J11
WM-010	2.533	3.609	2.283	2.380	2.978	2.800	1.891	1.495	3.793	3.680	3.859	1.783	3.500	2.457	2.747	EB2	E9	N4	M6	J9
WM-011	2.045	2.750	3.170	2.511	2.557	2.385	2.115	1.920	4.250	3.566	3.284	1.693	2.443	3.057	2.352	EB5	E9	N4	M6	J10
WM-012	2.483	2.843	2.841	2.500	2.404	2.481	1.674	1.921	4.551	3.957	3.157	1.787	2.506	3.360	1.886	EB6	E16	N12	M8	J8
WM-013	2.488	2.721	2.756	2.035	2.512	2.640	1.802	2.118	4.326	3.585*	3.476	1.872	2.553	3.209	1.965	EB6	E11	N9	M9	J7
WM-014	2.084	2.286	3.476	2.464	2.179	3.400	1.940	3.250	4.869	3.630	2.417	1.833	2.333	3.810	1.881	EB8	E9	N4	M5	J4
WM-015	2.128	2.628	3.512	2.977	2.198	3.043	2.314	2.174	4.302	3.943	2.588	1.882	2.384	3.314	2.395	EB6	E14	N5	M8	J10
WM-016	2.105	3.174*	3.256	1.849	2.523	4.125*	1.407	2.116	5.209*	4.018	2.616	1.616	3.059	3.081	1.953	EB9	E11	N8	M3	J4
WM-017	2.351	2.753	2.362	2.670	2.362	3.630*	1.670	2.340	4.468	4.106	3.096	1.946	3.117*	2.806	2.585	EB9	E14	N12	M6	J11
WM-018	1.890	2.444	2.700	1.780	2.143	3.519*	1.560	2.396	5.033	3.407	2.589	1.700	2.703	3.389	2.618	EB10	E4	N9	M6	J11
WM-019	2.261	3.500	2.283	2.446	2.793	3.929*	1.674	1.750	4.761	2.289	2.696	1.692	3.457	2.696	2.556	EB10	E13	N12	M6	J7
WM-020	2.420	2.432	2.693	2.227	2.205	2.593	1.818	2.239	4.580	4.280	3.239	1.875	2.568	3.068	2.227*	EB9	E16	N12	M9	J11
WM-021	2.176	3.200	2.659	1.671	2.329	3.958	1.424	1.976	5.294	2.220	2.536	1.988	3.259	3.000	2.405	EB10	E15	N1	M6	J3
WM-022	1.967	3.065	2.891	2.717	2.522	3.172	1.620	2.000	4.879	1.941	2.880	1.728	3.110	3.011	2.087	EB4	E8	N8	M8	J4
WM-023	1.890	1.758	2.867*	1.791	1.780	2.500	1.626	3.389	4.549	3.827	2.308	1.703	1.900	3.811	2.231	EB5	E11	N8	M9	J3
WM-024	2.427	1.939	3.646	3.610	1.901	2.280	2.220	2.890	3.951	3.269	2.585	2.183	1.817	3.780	1.914	EB10	E13	N12	M8	J11
WM-025	2.227	1.932	2.784	3.102	1.793	1.792	2.759	2.818	3.716	4.137	2.443	1.886	1.784*	3.489	2.682	EB9	E11	N8	M8	J11
WM-026	2.012	2.372	3.093	2.209	2.116	3.231	2.407	2.535	4.163	4.063	2.570	1.919	2.523	3.174	3.267	EB2	E11	N8	M4	J11
WM-028	2.525	2.563	3.200	2.550	2.538	2.636	2.200	2.638	4.263	2.022	3.075	2.388	2.413	3.550	2.900	EB4	E15	N4	M5	J7
WM-029	1.885	1.770	4.586*	2.494	1.655	2.821	1.609	2.667	5.207	3.760	2.276	1.655	1.828	3.701	2.046	EB8	E16	N12	M8	J7
WM-031	2.287	2.713	3.046	3.483	2.195	2.929	2.138	2.471	4.081	4.250	2.483	1.736	2.477	3.391	2.012	EB2	E12	N12	M8	J11
WM-032	1.816	1.851	2.644	3.253	1.884	3.120	1.885	3.686	4.517	3.933*	2.058	1.954	2.186	3.563	2.733	EB7	E13	N8	M5	J7
WM-033	1.725	1.756	3.846	2.231	1.835	2.815	1.780	2.511	4.556	4.143	2.244	1.516	1.846	3.582	1.900	EB6	E16	N5	M7	J9
WM-034	2.157	2.667	2.699	2.181	2.083	2.538	1.560	2.310	4.762	3.691	2.917	1.786	2.464	3.183	2.631	EB7	E7	N8	M4	J11
WM-035	2.593	3.835	2.165	3.033	3.429	2.963	1.725	1.901	4.484	3.357	3.088	1.923	3.396	2.758	2.527	EB7	E13	N9	M6	J8
WM-036	2.081	2.360	2.767	2.329	2.116	2.407	2.395	1.907	3.884	2.957	3.326	1.756	2.430*	2.826	3.244	EB3	E2	N5	M4	J3
WM-037	2.170	2.091	2.432	3.852	2.023	1.520	2.386	1.875	3.511	4.000	3.330	1.545	1.807*	3.125	2.686	EB5	E2	N5	M7	J10
WM-038	1.933	2.862	2.618	2.079	2.281	3.286	2.124	1.843	4.466	4.250	3.000	1.494	2.787	2.966	3.125	EB8	E14	N1	M9	J7
WM-039	2.116	2.024	2.430	3.294	1.930	1.640	2.593	1.953	3.541	4.333	3.244	1.512	1.756	3.024	3.059	EB9	E16	N5	M8	J1
WM-040	1.785	2.323	3.054	2.250	2.172	3.097	1.806	2.548	4.656	3.902	2.204	1.624	2.301	3.419	2.326	EB7	E10	N4	M1	J8
WM-041	2.190	2.800	2.141	3.141	2.624	2.560	1.729	1.750	4.141	2.089	2.612	1.718	2.635	2.976	2.412	EB10	E13	N8	M7	J6
WM-200	2.115	2.077	3.269	3.520	1.846	2.038	1.731	2.308	3.846	4.434	3.192	1.808	1.769	3.654	1.920	EB5	E16	N7	M7	J7
WM-201	1.727*	2.000	1.727*	2.783	1.913	2.739	1.364	2.636	4.636	4.020	2.435	1.739	1.739	3.130	2.261	EB10	E1	N10	M2	J2
WM-202	1.769	1.560	2.654	3.192	1.269	2.038	1.680	2.269	4.346	3.163	1.769	1.423	1.654	3.769*	2.000	EB10	E11	N9	M6	J6

WM-203	1.417	2.333	3.500	2.348	1.917	3.250	1.333	2.125	4.870	4.390	2.375	1.417	2.500	3.542	1.542	EB7	E10	N9	M6	J5
WM-204	1.433	1.933	3.200	1.900	1.586	3.500	1.483	2.100*	5.367	4.438	1.690	1.267	2.467	3.267	1.700	EB8	E12	N9	M5	J3
WM-205	1.840	3.042	3.800	2.320	2.200	3.520	1.542	1.600	5.000	4.429	1.960	1.440	2.600	3.040	1.920	EB10	E7	N10	M8	J5
WM-206	1.903	1.871	1.806	3.387	2.000	2.097	2.258*	2.677*	2.935*	3.839	2.032	1.774	2.000	3.258*	3.710	EB9	E14	N10	M6	J9
WM-207	1.478	1.625	4.125*	1.958	1.435	3.625	1.333	3.087	5.208	2.520	1.458	1.417	2.000	3.333	2.458	EB10	E11	N9	M5	J4
WM-208	1.929	1.679	2.655	3.724	1.828	2.179	2.034	3.241	3.310	3.732	2.000	1.966	1.655	3.586	1.655*	EB7	E14	N10	M5	J2
WM-209	2.222	1.571	3.536	3.607	1.821	1.821	2.143	2.357	3.607	3.980	2.269	1.893	1.821	3.536	1.714	EB9	E3	N7	M7	J7
WM-210	2.214	2.407	2.964	3.179	1.821	2.000	1.679	2.036	3.481	3.824	2.786	1.750	1.714	3.321	2.071	EB8	E13	N10	M8	J8
WM-211	1.786	1.536	3.464	2.069	1.464	2.690	1.759	1.966	4.607	3.808	1.621	1.586	1.897	3.172	3.345	EB5	E16	N10	M7	J9
WM-212	1.963*	3.333	3.704*	2.296	2.885	4.111	1.815	1.926	4.963	3.957	2.444	1.577	3.333	3.481	2.519	EB7	E1	N8	M9	J9
WM-213	1.760	1.960	3.708	3.000	1.560	3.000	1.920	3.826*	4.160	4.127	1.840	1.680	1.960	3.560	2.320	EB5	E11	N8	M7	J10
WM-214	1.800	1.680	3.120	3.600	1.720	2.480	2.250	3.400	3.667*	4.132	1.880	2.040	1.680	3.720	2.240	EB6	E16	N12	M8	J11
WM-215	1.964	2.071	1.893	3.000	2.071	2.071	1.786	1.815	4.185	3.432	3.857	1.929	2.000	3.107	2.429	EB10	E15	N9	M8	J4
WM-216	2.654	2.115	2.654	2.962	2.308	2.077	2.000	1.615	3.615	3.841	3.000	2.038	2.077	3.192	2.192	EB8	E9	N10	M4	J10
WM-217	2.500	3.462	3.077	4.231*	2.269	2.808	2.769*	1.846	3.462*	2.813	2.538	2.154	2.846	3.115	2.962	EB10	E16	N10	M8	J8
WM-218	1.536	2.071*	2.857	1.857	1.571	2.500	1.357	1.889	4.393	2.596	2.071*	1.464	1.778	3.107	1.893*	EB3	E8	N9	M9	J1
WM-219	1.708	2.000*	2.958	2.348	1.458	2.833	1.792	1.750	4.583	4.096	3.167	1.708	1.750	3.250	1.625	EB10	E12	N7	M9	J8
WM-220	1.926	3.741	2.519	1.852	2.444	4.615	1.407	2.308	5.074	3.792	2.000	1.852	4.385	2.481	3.407	EB7	E7	N10	M8	J8
WM-221	1.593	3.148	3.154	1.704	2.296	4.259	1.370	1.889	4.926	4.390	2.538	1.519	2.519	3.556	1.926	EB7	E10	N10	M9	J8
WM-222	3.000	2.750	2.345	2.207	2.517	2.571	2.138*	1.655	4.071	3.455	2.793	2.000	2.586	2.759	2.724	EB10	E12	N7	M2	J8
WM-223	1.583	2.880	3.080	1.120	2.120	4.360	1.000	1.960	5.480	3.170	2.542	1.360	3.083	3.000	2.040	EB8	E15	N3	M5	J5
WM-224	2.222	3.143	2.464	1.786*	2.179	4.179*	1.893	1.571	4.750*	4.107	2.143	1.536	3.643	2.821	4.286	EB9	E14	N7	M8	J8
WM-225	1.500	1.926	3.036	1.893	1.407	3.107	1.357	2.679	4.607	4.385	1.857	1.393	2.000	3.704	2.036	EB8	E16	N10	M8	J9
WM-227	1.773	2.545	2.435	2.000	1.955	3.609	1.739	1.826	4.739	1.811*	2.522	1.609	2.348	3.087	1.913	EB2	E6	N7	M9	J7
WM-228	1.769	2.556	1.889	2.346	2.259	3.148	1.815	2.115	4.423	1.636	2.519	1.630	2.577	3.000	3.593	EB10	E8	N1	M6	J2
WM-229	1.792	2.696	2.792	2.083	1.875	2.625	1.250	1.913	4.125	4.000	2.167	1.417	2.292	2.958	1.875	EB10	E12	N7	M7	J3
WM-230	1.815	1.786	3.143	3.964	2.000	2.679	1.852	3.214	3.821	4.273	1.929	1.500	2.036	3.393	2.036	EB5	E9	N7	M9	J6
WM-231	2.000	2.172	3.643*	4.069	1.655	2.607	1.862	2.310	4.241	2.235	2.586	1.483	2.138	3.276	1.897	EB3	E6	N6	M7	J6
WM-232	2.667	3.538	2.593	2.704*	2.926	2.593	1.741	1.889	4.370	3.625	3.407	1.852	3.037	2.778	2.704	EB10	E12	N6	M7	J11
WM-233	2.208	2.391	2.125	2.000	2.375	3.333	1.833	2.375	4.542	1.571	2.292	1.917	2.500	3.000	2.917	EB4	E15	N11	M7	J10
WM-234	1.862	2.862	2.552	2.724	2.000	3.107	1.586	2.138	4.517	4.260	2.172	1.310	2.517	2.862	1.897	EB4	E5	N11	M2	J2
WM-235	1.429	3.000	3.357	2.464	2.037	3.074	1.571	1.679	4.321	4.250	2.074	1.357	3.000	2.571	1.679	EB1	E5	N6	M7	J9
WM-236	1.692	1.500	2.038	3.808	1.308	1.560*	2.769	1.923	2.840	3.170	2.923	1.231	1.538	3.000	2.769	EB2	E4	N6	M4	J10
WM-237	1.966	2.552	2.517	2.069	2.172	2.724	1.679	1.793	4.286	3.792	2.552	1.643	2.414	3.000	2.414	EB3	E6	N5	M8	J8
WM-238	1.667	2.364	3.273	2.182	2.136	3.318	1.500	2.409	4.591	3.712	1.773	1.429	2.909	3.000	2.136	EB4	E7	N11	M7	J11
WM-239	2.321*	2.000	2.964	4.286	2.214	2.179	2.071	2.321	3.786	3.778	2.393	1.893	2.107	3.286	1.964	EB4	E9	N6	M4	J7
WM-240	3.385*	2.200	3.346	2.192	2.038	2.731	1.920	1.760	4.500	3.489	4.154	1.731*	2.231	3.500	2.654	EB8	E7	N6	M7	J10
WM-241	2.000*	3.462*	3.000	1.889	2.037	4.037	2.000	2.259	4.704	4.043	2.259*	1.577	3.000	3.111	2.926	EB7	E10	N11	M8	J9
WM-242	1.759	2.571	4.034	2.310	2.724	2.828	1.759	1.724	4.214	3.255	2.241	1.345	2.143	3.138	2.310	EB1	E16	N6	M7	J5
WM-243	1.880	3.200	3.160	1.840	2.375	3.880	1.760	1.760	4.652	2.410	2.200	1.600	3.560	2.560	3.360	EB7	E15	N3	M9	J11
WM-244	1.538	2.037	3.037	1.852	1.680	3.481	1.692	2.815	5.111	3.386	1.778	1.778	2.370	2.889	2.370	EB4	E5	N3	M3	J6
WM-245	2.586*	2.931	3.357	2.143	2.714	2.862	1.345	1.448	4.621	3.956	3.414	1.379	2.759	2.828	1.931	EB8	E15	N11	M9	J9
WM-247	2.040	2.269	2.720	2.462	2.038	2.615	1.769	2.577	3.923	2.765	2.458	1.769	2.077	3.231	2.154	EB4	E15	N6	M9	J9
WM-248	2.000	2.000	2.846	1.731	2.000	2.962	1.360	2.308	4.462	4.040	2.500	1.923	2.385	3.231	2.038	EB5	E5	N7	M2	J10
WM-249	2.481	3.037	2.407	1.556	2.407	3.333	1.222	1.333*	4.148	4.100	3.222	1.538	3.407	2.308*	2.889	EB6	E15	N9	M9	J6
WM-250	2.167	1.880	4.120	3.720	2.040	3.640	1.560	1.875	4.680	1.250	2.680	1.640	2.000	3.360	2.240	EB10	E3	N6	M7	J9
WM-251	2.040	2.080	2.600	2.080	1.960	2.560	1.680	3.200	4.320	4.038*	2.480	1.792	1.920	3.400*	2.480	EB10	E15	N11	M4	J3
WM-252	1.778	2.296	3.111	2.000	2.074	3.037	1.370	2.704	5.148	2.222	1.889	1.741	1.963	3.889*	2.222	EB4	E15	N3	M5	J5
WM-253	1.538	1.692	3.500	3.077	1.769	2.760	1.760	3.269	3.846	2.049	1.731	1.654	2.000	3.154	2.346	EB3	E9	N11	M3	J3
WM-254	1.308	2.077	3.154	2.077	1.615	3.231	1.231	2.538	4.808	4.149	1.269*	1.154	2.000	3.231	1.308	EB10	E16	N12	M9	J4
WM-255	1.444	2.250	2.259	1.571	1.821	3.214	1.643	2.571	4.714	1.769	2.143	1.393	2.750	2.893	3.000	EB10	E15	N11	M9	J9
WM-256	1.769	2.385	2.269	2.615	1.962	3.231	1.640	2.346	4.385	2.214	1.960	1.923	2.385	3.000	1.769	EB10	E11	N12	M6	J6
WM-257	1.926	1.333	3.741	2.280	1.185	2.185	1.815	2.519	4.346	3.082	2.519	1.741	1.556	3.923	2.667	EB5	E12	N12	M3	J10
WM-258	1.222	1.630	2.148	1.778	1.852	3.231	1.148*	3.074	4.667	4.244	1.593	1.519	2.000	3.333	2.185	EB6	E16	N11	M8	J9
Mean	2.006	2.425	2.961	2.554	2.094	2.943	1.797	2.280	4.400	3.501	2.521	1.692	2.401	3.210	2.390					
SD	0.370	0.587	0.602	0.698	0.407	0.664	0.364	0.524	0.530	0.819	0.564	0.227	0.569	0.338	0.536					

Participants responded on a 1–7 Likert scale (1 = Not at all, 7 = Extremely) except for Prototypic, that was responded to on a 1-5 Likert scale. Some observations with abnormal studentized residuals were considered outliers and removed from the models (marked with an asterisk in the Table S1)

Table S2. Skewness, Kurtosis and Kolmogorov-Smirnov test for residuals of each model.

			Skewness		Kurtosis		Kolmogorov-Smirnov[a]		
Residuals	M	SD	Statistic	Std. Error	Statistic	Std. Error	Statistic	df	Sig.
Afraid	0.000	.132	-.115	.258	-.641	.511	.056	87	.200*
Angry	0.000	.253	-.218	.255	-.051	.506	.054	89	.200*
Attractive	0.000	.212	.323	.258	-.632	.511	.069	87	.200*
Baby-faced	0.000	.306	.137	.254	-.010	.530	.073	90	.200*
Disgusted	0.000	.253	-.106	.250	-.202	.495	.058	93	.200*
Dominant	0.000	.280	-.069	.258	-.173	.511	.051	87	.200*
Feminine	0.000	.136	.188	.257	-.349	.508	.068	88	.200*
Happy	0.000	.255	-.043	.255	-.603	.506	.077	89	.200*
Masculine	0.000	.199	.118	.257	-.350	.508	.066	88	.200*
Prototypic	0.000	.260	.033	.255	-.323	.506	.054	89	.200*
Sad	0.000	.275	-.193	.254	-.306	.503	.088	90	.200*
Surprised	0.000	.109	.011	.251	-.310	.498	.054	92	.200*
Threatening	0.000	.270	-.149	.255	-.366	.506	.074	89	.200*
Trustworthy	0.000	.130	.129	.257	-.503	.508	.056	88	.200*
Unusual	0.000	.250	.227	.254	-.860	.503	.078	90	.200*

[a] Lilliefors Significance Correction

*This is a lower bound of the true significance

Figure S1. Predicted vs. observed values, residuals on predicted values and normal probability of residuals of each model.

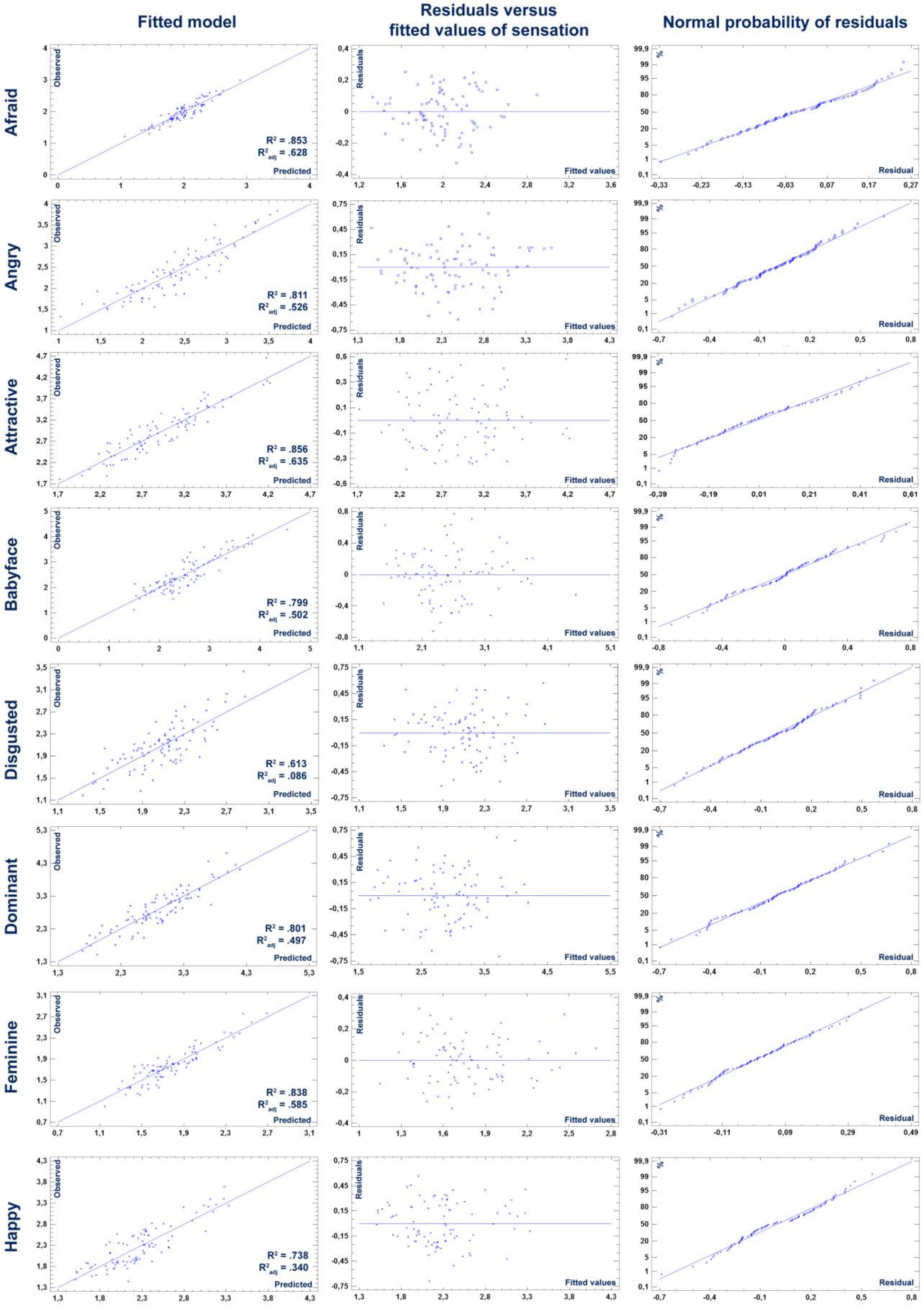

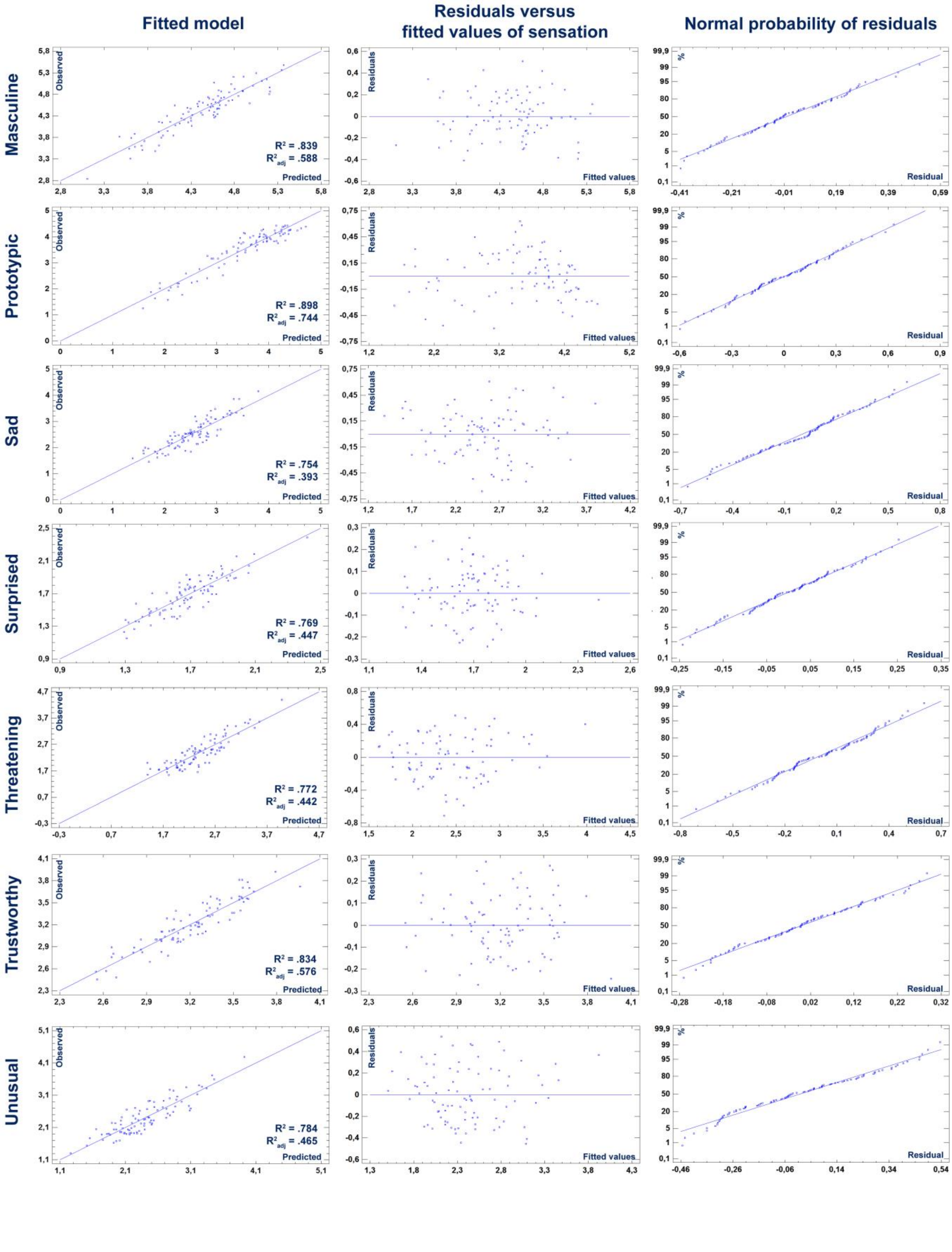

Fitted model
Residuals versus fitted values of sensation
Normal probability of residuals
Masculine
R^2 = .839
R^2_{adj} = .588
Prototypic
R^2 = .898
R^2_{adj} = .744
Sad
R^2 = .754
R^2_{adj} = .393
Surprised
R^2 = .769
R^2_{adj} = .447
Threatening
R^2 = .772
R^2_{adj} = .442
Trustworthy
R^2 = .834
R^2_{adj} = .576
Unusual
R^2 = .784
R^2_{adj} = .465
Observed
Predicted
Residuals
Fitted values
%
Residual

Figure S2. Effect size of facial features on observed facial traits as a percentage of all the effects by facial feature.

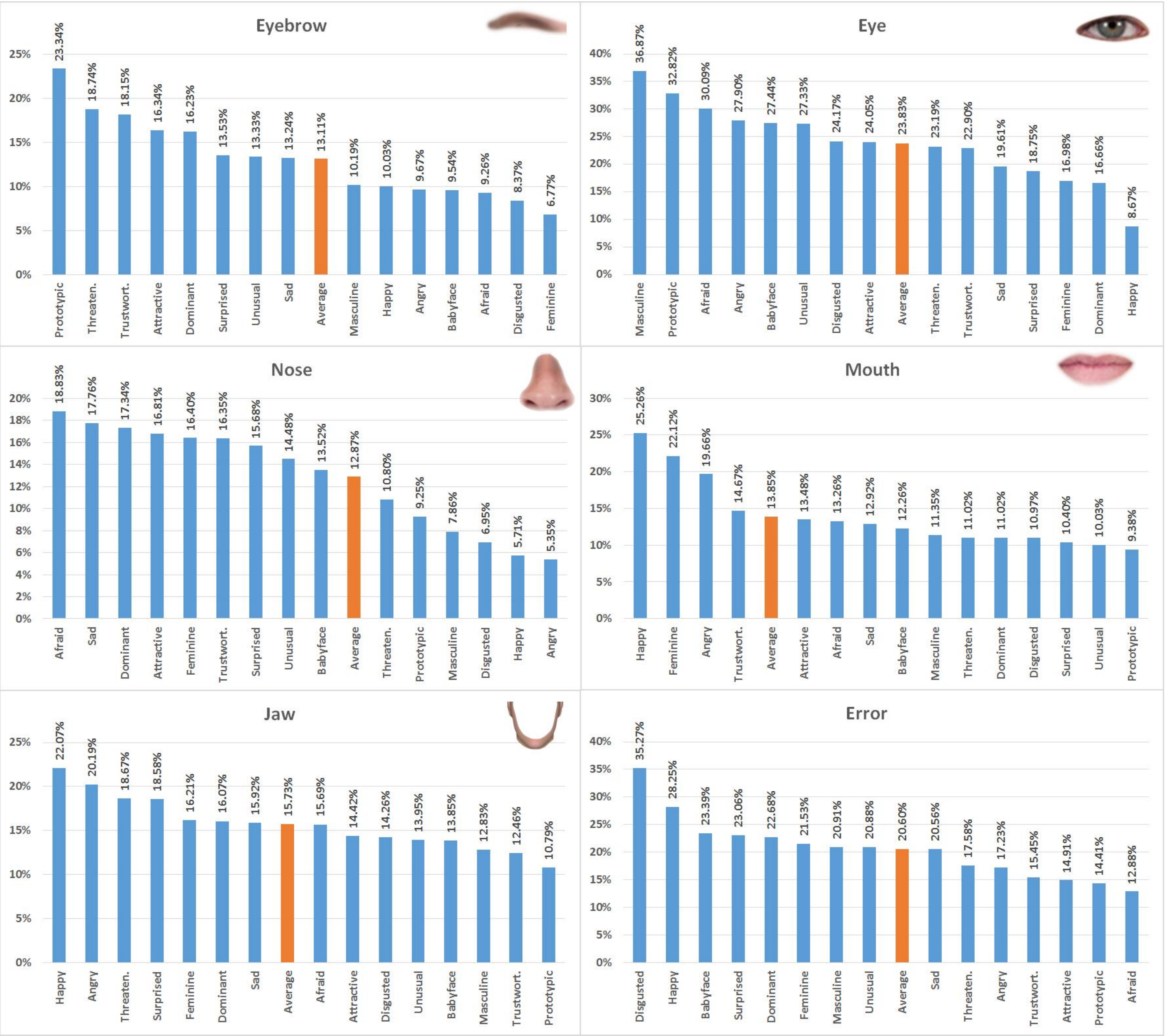

Table S3. Results of univariate ANOVA analysis for the effect of facial features on facial traits

Test of Between-subjects Effects. Dependent Variable: **Afraid**

Source	Type III Sum of Squares	df	Mean Square	F	Sig.	Partial Eta Squared
Corrected Model	8.671[a]	52	.167	3.795	.000	.853
Intercept	52.131	1	52.131	1186.415	.000	.972
Eyebrow	1.074	9	.119	2.717	.017	.418
Eye	3.491	14	.249	5.675	.000	.700
Nose	2.185	11	.199	4.520	.000	.594
Mouth	1.538	8	.192	4.375	.001	.507
Jaw	1.821	10	.182	4.144	.001	.549
Error	1.494	34	.044			
Total	352.567	87				
Corrected Total	10.165	86				

a. R Squared = .853 (Adjusted R Squared = .628) Removed observations: WM-201, WM-212, WM-239, WM-240, WM-241, WM-245

Test of Between-subjects Effects. Dependent Variable: **Angry**

Source	Type III Sum of Squares	df	Mean Square	F	Sig.	Partial Eta Squared
Corrected Model	24.131[a]	53	.455	2.839	.001	.811
Intercept	71.005	1	71.005	442.773	.000	.927
Eyebrow	3.150	9	.350	2.182	.048	.359
Eye	9.090	15	.606	3.779	.001	.618
Nose	1.743	11	.158	.988	.475	.237
Mouth	6.407	8	.801	4.994	.000	.533
Jaw	6.579	10	.658	4.102	.001	.540
Error	5.613	35	.160			
Total	548.367	89				
Corrected Total	29.744	88				

a. R Squared = .811 (Adjusted R Squared = .526) Removed observations: WM-016, WM-218, WM-219, WM-241

Test of Between-subjects Effects. Dependent Variable: **Attractive**

Source	Type III Sum of Squares	df	Mean Square	F	Sig.	Partial Eta Squared
Corrected Model	22.863[a]	52	.440	3.876	.000	.856
Intercept	119.978	1	119.978	1057.803	.000	.969
Eyebrow	4.226	9	.470	4.140	.001	.523
Eye	6.221	14	.444	3.918	.001	.617
Nose	4.347	11	.395	3.484	.002	.530
Mouth	3.486	8	.436	3.842	.003	.475
Jaw	3.729	10	.373	3.288	.005	.492
Error	3.856	34	.113			
Total	772.604	87				
Corrected Total	26.720	86				

a. R Squared = .856 (Adjusted R Squared = .635) Removed observations: WM-023, WM-029, WM-201, WM-207, WM-212, WM-231

Test of Between-subjects Effects. Dependent Variable: **Baby-faced**

Source	Type III Sum of Squares	df	Mean Square	F	Sig.	Partial Eta Squared
Corrected Model	33.038[a]	53	.623	2.692	.001	.799
Intercept	88.012	1	88.012	380.075	.000	.913
Eyebrow	3.399	9	.378	1.631	.143	.290
Eye	9.781	15	.652	2.816	.006	.540
Nose	4.820	11	.438	1.892	.074	.366
Mouth	4.368	8	.546	2.358	.038	.344
Jaw	4.937	10	.494	2.132	.047	.372
Error	8.336	36	.232			
Total	622.973	90				
Corrected Total	41.374	89				

a. R Squared = .799 (Adjusted R Squared = .502) Removed observations: WM-217, WM-224, WM-232

Test of Between-subjects Effects. Dependent Variable: **Disgusted**

Source	Type III Sum of Squares	df	Mean Square	F	Sig.	Partial Eta Squared
Corrected Model	9.321[a]	53	.176	1.163	.313	.613
Intercept	67.439	1	67.439	446.148	.000	.920
Eyebrow	1.399	9	.155	1.028	.435	.192
Eye	4.040	15	.269	1.782	.074	.407
Nose	1.161	11	.106	.698	.732	.165
Mouth	1.834	8	.229	1.517	.183	.237
Jaw	2.383	10	.238	1.576	.150	.288
Error	5.895	39	.151			
Total	422.830	93				
Corrected Total	15.216	92				

a. R Squared = .613 (Adjusted R Squared = .086) Removed observations: None

Test of Between-subjects Effects. Dependent Variable: **Dominant**

Source	Type III Sum of Squares	df	Mean Square	F	Sig.	Partial Eta Squared
Corrected Model	27.084[a]	52	.521	2.631	.002	.801
Intercept	131.823	1	131.823	665.939	.000	.951
Eyebrow	4.817	9	.535	2.704	.017	.417
Eye	4.943	14	.353	1.784	.084	.423
Nose	5.147	11	.468	2.364	.027	.433
Mouth	3.269	8	.409	2.064	.068	.327
Jaw	4.769	10	.477	2.409	.027	.415
Error	6.730	34	.198			
Total	768.155	87				
Corrected Total	33.814	86				

a. R Squared = .801 (Adjusted R Squared = .497) Removed observations: WM-016, WM-017, WM-018, WM-019, WM-224, WM-236

Test of Between-subjects Effects. Dependent Variable: **Feminine**

Source	Type III Sum of Squares	df	Mean Square	F	Sig.	Partial Eta Squared
Corrected Model	8.262[a]	53	.156	3.315	.000	.838
Intercept	37.230	1	37.230	791.677	.000	.959
Eyebrow	.503	9	.056	1.189	.333	.239
Eye	1.261	15	.084	1.788	.079	.441
Nose	1.218	11	.111	2.354	.028	.432
Mouth	1.643	8	.205	4.366	.001	.507
Jaw	1.204	10	.120	2.560	.020	.430
Error	1.599	34	.047			
Total	287.184	88				
Corrected Total	9.861	87				

a. R Squared = .838 (Adjusted R Squared = .585) Removed observations: WM-006, WM-206, WM-217, WM-222, WM-258

Test of Between-subjects Effects. Dependent Variable: **Happy**

Source	Type III Sum of Squares	df	Mean Square	F	Sig.	Partial Eta Squared
Corrected Model	16.040[a]	53	.303	1.857	.027	.738
Intercept	86.205	1	86.205	528.866	.000	.938
Eyebrow	2.026	9	.225	1.381	.234	.262
Eye	1.751	15	.117	.716	.752	.235
Nose	1.154	11	.105	.644	.779	.168
Mouth	5.102	8	.638	3.912	.002	.472
Jaw	4.457	10	.446	2.734	.013	.439
Error	5.705	35	.163			
Total	480.826	89				
Corrected Total	21.745	88				

a. R Squared = .738 (Adjusted R Squared = .340) Removed observations: WM-204, WM-206, WM-213, WM-249

Test of Between-subjects Effects. Dependent Variable: **Masculine**

Source	Type III Sum of Squares	df	Mean Square	F	Sig.	Partial Eta Squared
Corrected Model	17.981[a]	53	.339	3.341	.000	.839
Intercept	279.389	1	279.389	2751.349	.000	.988
Eyebrow	1.682	9	.187	1.840	.097	.328
Eye	6.088	15	.406	3.997	.000	.638
Nose	1.298	11	.118	1.162	.348	.273
Mouth	1.874	8	.234	2.307	.043	.352
Jaw	2.119	10	.212	2.087	.054	.380
Error	3.453	34	.102			
Total	1742.237	88				
Corrected Total	21.433	87				

a. R Squared = .839 (Adjusted R Squared = .588) Removed observations: WM-016, WM-206, WM-214, WM-217, WM-224

Test of Between-subjects Effects. Dependent Variable: **Prototypic**

Source	Type III Sum of Squares	df	Mean Square	F	Sig.	Partial Eta Squared
Corrected Model	52.363[a]	53	.988	5.832	.000	.898
Intercept	194.730	1	194.730	1149.487	.000	.970
Eyebrow	9.601	9	1.067	6.297	.000	.618
Eye	13.502	15	.900	5.313	.000	.695
Nose	3.806	11	.346	2.043	.054	.391
Mouth	3.859	8	.482	2.848	.015	.394
Jaw	4.440	10	.444	2.621	.017	.428
Error	5.929	35	.169			
Total	1153.701	89				
Corrected Total	58.292	88				

a. R Squared = .898 (Adjusted R Squared = .744) Removed observations: WM-013, WM-032, WM-227, WM-251

Test of Between-subjects Effects. Dependent Variable: **Sad**

Source	Type III Sum of Squares	df	Mean Square	F	Sig.	Partial Eta Squared
Corrected Model	20.691[a]	53	.390	2.087	.011	.754
Intercept	93.549	1	93.549	500.203	.000	.933
Eyebrow	4.336	9	.482	2.576	.021	.392
Eye	6.421	15	.428	2.289	.021	.488
Nose	5.816	11	.529	2.827	.009	.463
Mouth	4.232	8	.529	2.828	.015	.386
Jaw	5.213	10	.521	2.787	.012	.436
Error	6.733	36	.187			
Total	609.410	90				
Corrected Total	27.424	89				

a. R Squared = .754 (Adjusted R Squared = .393) Removed observations: WM-218, WM-241, WM-254

Test of Between-subjects Effects. Dependent Variable: **Surprised**

Source	Type III Sum of Squares	df	Mean Square	F	Sig.	Partial Eta Squared
Corrected Model	3.629[a]	53	.068	2.385	.003	.769
Intercept	41.623	1	41.623	1449.983	.000	.974
Eyebrow	.640	9	.071	2.476	.025	.370
Eye	.887	15	.059	2.060	.036	.448
Nose	.742	11	.067	2.350	.025	.405
Mouth	.492	8	.061	2.141	.055	.311
Jaw	.879	10	.088	3.062	.006	.446
Error	1.091	38	.029			
Total	267.948	92				
Corrected Total	4.720	91				

a. R Squared = .769 (Adjusted R Squared = .447) Removed observations: WM-240

Test of Between-subjects Effects. Dependent Variable: **Threatening**

Source	Type III Sum of Squares	df	Mean Square	F	Sig.	Partial Eta Squared
Corrected Model	21.588[a]	52	.415	2.338	.004	.772
Intercept	77.331	1	77.331	435.485	.000	.924
Eyebrow	6.814	9	.757	4.263	.001	.516
Eye	8.434	14	.602	3.393	.002	.569
Nose	3.926	11	.357	2.010	.057	.380
Mouth	4.008	8	.501	2.821	.015	.385
Jaw	6.787	10	.679	3.822	.001	.515
Error	6.393	36	.178			
Total	541.836	89				
Corrected Total	27.981	88				

a. R Squared = .772 (Adjusted R Squared = .442) Removed observations: WM-017, WM-025, WM-036, WM-037

Test of Between-subjects Effects. Dependent Variable: **Trustworthy**

Source	Type III Sum of Squares	df	Mean Square	F	Sig.	Partial Eta Squared
Corrected Model	7.433[a]	53	.140	3.228	.000	.834
Intercept	151.978	1	151.978	3497.679	.000	.990
Eyebrow	1.735	9	.193	4.436	.001	.540
Eye	2.189	15	.146	3.358	.002	.597
Nose	1.563	11	.142	3.270	.004	.514
Mouth	1.402	8	.175	4.033	.002	.487
Jaw	1.191	10	.119	2.740	.014	.446
Error	1.477	34	.043			
Total	911.772	88				
Corrected Total	8.911	87				

a. R Squared = .834 (Adjusted R Squared = .576) Removed observations: WM-202, WM-206, WM-249, WM-251, WM-252

Test of Between-subjects Effects. Dependent Variable: **Unusual**

Source	Type III Sum of Squares	df	Mean Square	F	Sig.	Partial Eta Squared
Corrected Model	20.081[a]	53	.379	2.461	.003	.784
Intercept	519.144	1	519.144	3372.334	.000	.989
Eyebrow	3.538	9	.393	2.553	.022	.390
Eye	7.254	15	.484	3.142	.002	.567
Nose	3.844	11	.349	2.270	.032	.410
Mouth	2.663	8	.333	2.162	.055	.325
Jaw	3.703	10	.370	2.406	.026	.401
Error	5.542	36	.154			
Total	544.768	90				
Corrected Total	25.623	89				

a. R Squared = .784 (Adjusted R Squared = .465) Removed observations: WM-020, WM-208, WM-218,
